# Gene Correction Enhances Dopaminergic Cell Therapy in a Nonhuman Primate Model of Parkinson's Disease

**DOI:** 10.1002/advs.76394

**Published:** 2026-07-06

**Authors:** Qing Yan, Chongchong Xu, Jiangmei Gao, Pu Wang, Qingling Wu, Ying Jin, Binyan Lu, Mu Li, Mingting Shao, Bihai Li, Zhenghui Su, Yijng Zhang, Jianhuan Chen, Haojie An, Mengyao Huang, Xiaoji Zhuang, Yuhui Shen, Fenglin Wang, Nana Xu, Yiyan Liu, Lei Tang, Xiuling Zhong, Minyan Zhong, Jiecong Chen, Zhenhuang Wang, Xingrong Luo, Sheng Liu, RuiFeng Liu, Ling Zhang, Junhua Rao

**Affiliations:** ^1^ Guangdong Key Laboratory of Animal Conservation and Resource Utilization Guangdong Public Laboratory of Wild Animal Conservation and Utilization Institute of Zoology Guangdong Academy of Sciences Guangzhou Guangdong China; ^2^ Zhuzhou Central Hospital Zhuzhou Hunan China; ^3^ Guangzhou Regenverse Therapeutics Co., Ltd Guangzhou China; ^4^ Chengdu Wenjiang District People's Hospital Chengdu China; ^5^ State Key Laboratory of Ophthalmology Zhongshan Ophthalmic Center Sun Yat‐sen University Guangdong Provincial Key Laboratory of Ophthalmology and Visual Science Guangzhou China; ^6^ Laboratory of Genomic and Precision Medicine Wuxi School of Medicine Jiangnan University Wuxi Jiangsu China; ^7^ The Third Affiliated Hospital Sun Yat‐sen University Guangzhou China

**Keywords:** cell therapy, CRISPR/Cas9, dopaminergic neurons, gene correction, induced pluripotent stem cells, nonhuman primate model, parkinson's disease

## Abstract

Human induced pluripotent stem cell (iPSC)‐derived dopaminergic progenitors offer a promising strategy for cell replacement in Parkinson's disease (PD), yet their long‐term efficacy and safety in primates remain underexplored. Here, we generated isogenic iPSC lines from a PD patient carrying compound LRRK2 mutations (R50H and M2397T) and used CRISPR/Cas9 and prime editing to correct these variants. Both mutant and gene‐corrected lines were differentiated into midbrain dopaminergic progenitors (DA‐NPCs), characterized in vitro, and transplanted bilaterally into the striatum of MPTP‐lesioned cynomolgus monkeys. Over 18 months, grafted cells survived, expressed TH and GIRK2, and exhibited mature electrophysiological properties. [^1^ ^8^F] DOPA PET imaging revealed restored dopamine synthesis in both grafting groups, with no statistically significant differences observed between mutant and gene‐corrected groups. Behavioral assessments showed sustained motor improvements and enhanced exploratory behavior. No tumor formation or adverse astrocytic response was observed, and systemic safety markers remained normal. These findings demonstrate that both mutant and corrected DA‐NPCs integrate and function in the primate brain. Our results support the feasibility of personalized regenerative therapies for genetic PD.

## Graphical abstract

LRRK2‐mutant induced pluripotent stem cells (iPSCs) were derived from a patient with Parkinson's disease (PD). Using CRISPR/Cas9–mediated gene editing, the pathogenic LRRK2 mutations were precisely corrected, and isogenic dopaminergic neural progenitor cells (DA‐NPCs) were subsequently generated. These gene‐corrected DA‐NPCs were stereotactically transplanted into the striatum of MPTP‐lesioned PD monkeys. Grafted cells demonstrated long‐term survival—up to 18 months post‐transplantation—and robustly differentiated into tyrosine hydroxylase–positive (TH^+^) and microtubule‐associated protein 2–positive (MAP2^+^) neurons in vivo. Functional assessments revealed significant restoration of dopaminergic neurotransmission and sustained improvement in motor behavior in treated animals, with no clinical evidence of tumorigenicity, immunogenicity, or other adverse effects. These findings collectively support the therapeutic potential of autologous, genetically corrected iPSC‐derived DA‐NPCs for monogenic forms of Parkinson's disease.

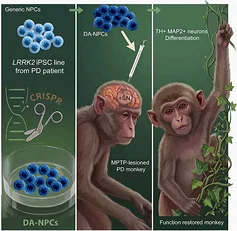

## 1 Introduction

Parkinson's disease (PD) is a progressive neurodegenerative disorder characterized by the loss of midbrain dopaminergic neurons in the substantia nigra, leading to motor impairments such as bradykinesia, rigidity, and tremor, as well as a range of non‐motor symptoms including apathy, depression, and cognitive decline [1, 2, 3, 4]. While current pharmacological treatments offer symptomatic relief, they do not halt disease progression or restore lost neurons, and long‐term use is often complicated by motor fluctuations and dyskinesias [1, 2, 3, 4]. Cell replacement therapy using dopaminergic progenitors offers a potentially disease‐modifying approach by reconstituting the nigrostriatal circuitry [5, 6, 7, 8, 9].

Human induced pluripotent stem cells (iPSCs) have emerged as a powerful source for generating midbrain dopaminergic neurons suitable for transplantation [5, 6, 7, 8, 9, 10, 11, 12, 13, 14, 15, 16]. They provide an ethically viable, renewable platform and can be derived autologously from patients, potentially eliminating immune rejection [17, 18, 19, 20, 21]. However, the use of iPSCs in PD therapy raises important questions, particularly in patients with familial mutations such as those in the LRRK2 gene, one of the common genetic contributors to both familial and sporadic PD [22, 23, 24, 25, 26, 27, 28]. Whether these mutations compromise the differentiation potential, function, or safety of patient‐derived DA progenitors remains poorly understood.

Recent advances in genome editing, including CRISPR/Cas9 and prime editing technologies [29, 30, 31, 32, 33, 34, 35], now allow precise correction of disease‐causing mutations in patient‐derived iPSCs, enabling the generation of isogenic controls to dissect genotype‐phenotype relationships and evaluate therapeutic efficacy. The necessity of gene correction in autologous iPSC‐based therapies for genetic PD remains an actively debated topic. A common counterargument posits that disease‐related phenotype mutations may take decades to manifest in transplanted cells, raising the question of whether early‐stage correction is clinically essential [24, 36, 37]. However, emerging evidence suggests that PD‐associated mutations, including LRRK2 variants, can alter cellular stress responses, mitochondrial function, and synaptic activity even at early developmental stages, potentially impacting graft robustness, function, or long‐term stability [37, 38, 39]. This ongoing debate underscores the critical need for empirical evaluation in clinically relevant models, which forms a core motivation for the present study. Although previous studies have demonstrated short‐term survival and partial functional integration of iPSC‐derived dopaminergic neurons in rodent models [40, 41, 42, 43, 44], comprehensive long‐term studies in non‐human primates, necessary to model the human brain environment and assess clinical relevance, are still few [43, 45, 46, 47, 48, 49]. Existing long‐term investigations in non‐human primates (NHPs) have primarily utilized wild‐type or allogeneic cell sources, with limited focus on patient‐specific genetic backgrounds or direct comparison of isogenic mutant versus gene‐corrected grafts within the same donor [43, 46, 47, 48, 49].

In this study, we generated isogenic iPSC lines from a PD patient carrying compound heterozygous LRRK2 mutations and corrected these mutations using CRISPR/Cas9 and prime editing. We differentiated both mutant and gene‐corrected lines into dopaminergic progenitors and transplanted them into the striatum of MPTP‐lesioned cynomolgus monkeys. Using longitudinal behavioral tracking, neuroimaging, electrophysiology, and histopathology over 18 months, we evaluated the safety, survival, functional integration, and therapeutic impact of the grafts. Our findings provide crucial insights into the feasibility and translational potential of gene‐corrected autologous iPSC‐derived cell therapy for PD.

## 2 Results

### 2.1 Generation and Characterization of LRRK2 Mutant and Corrected iPSC Lines

To model Parkinson's disease (PD), we established induced pluripotent stem cell (iPSC) lines from a PD patient harboring compound heterozygous mutations in the LRRK2 gene (R50H and M2397T). These mutations were corrected using a combination of CRISPR/Cas9 and PE3 prime editing [34]. Sanger sequencing confirmed precise editing at both locations, eliminating the pathogenic variants without introducing off‐target mutations or genomic instability (Figure 1A). All iPSC lines, including both LRRK2 mutant and isogenic gene‐corrected lines, retained classical features of pluripotency. Immunocytochemistry confirmed the expression of key pluripotency markers SSEA4 and NANOG (Figure S1A), and flow cytometry demonstrated high expression of SSEA4 and TRA‐1‐60 across all lines (Figure S1B), indicating robust reprogramming and maintenance of stemness. Trilineage differentiation assays further demonstrated the developmental potential of these lines. Immunofluorescence showed efficient differentiation into βIII‐Tubulin+ ectodermal neurons, α‐SMA+ mesodermal cells, and AFP+ endodermal cells (Figure S1C), confirming their ability to give rise to all three germ layers. Midbrain‐specific lineage commitment was assessed by directed neural induction, with flow cytometry revealing a high proportion of cells co‐expressing LMX1 and FOXA2 (Figure S1D), markers associated with floor plate‐derived midbrain dopaminergic progenitors.

**FIGURE 1:**
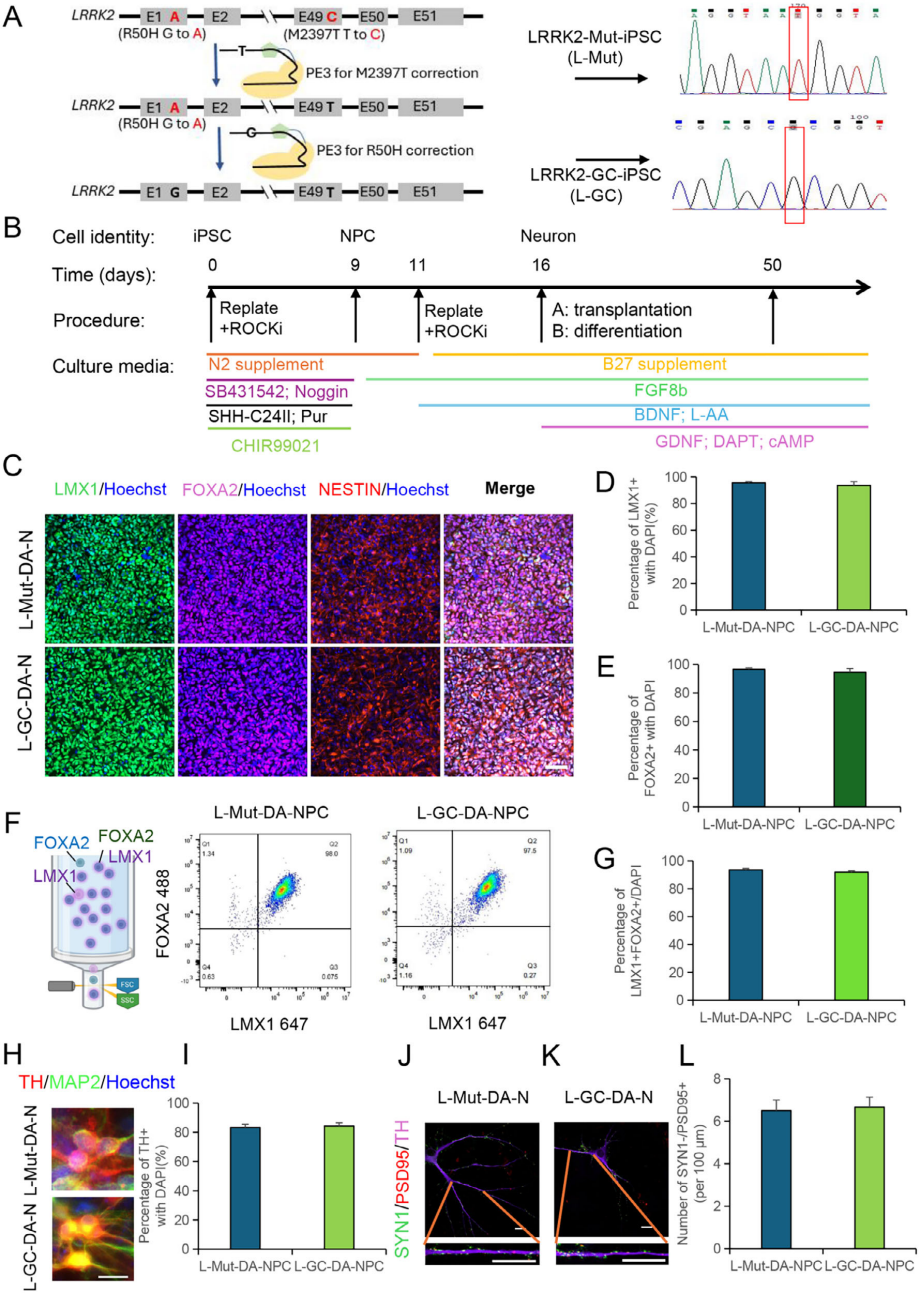
Differentiation of iPSC lines into dopamine neurons. (A) Schematic representation of the LRRK2 locus in a Parkinson's disease (PD) patient‐derived iPSC line harboring two mutations (R50H, M2397T). The correction strategy using CRISPR/Cas9 and prime editing (PE3) is shown, with successful editing confirmed by Sanger sequencing. (B) Overview of the differentiation protocol for generating dopamine neurons (DA neurons) from iPSCs. Key supplements and factors used at each stage are indicated. (C) Representative immunofluorescence images of day 16 dopamine neural progenitor cells (DA‐NPCs), showing co‐expression of LMX1 (green), FOXA2 (magenta), and NESTIN (red). Nuclei are counterstained with Hoechst. Scale bar, 50 µm. (D, E) Quantification of LMX1^+^/Hoechst^+^ and FOXA2^+^/Hoechst^+^ cells on day 16. Data are shown as mean ± SD. (F) Representative flow cytometry plots showing co‐expression of LMX1 and FOXA2 in day 16 DA‐NPCs. (G) Quantification of LMX1^+^FOXA2^+^/Hoechst^+^ triple‐positive DA‐NPCs. (H) Immunofluorescence staining of day 50 differentiated neurons showing co‐expression of tyrosine hydroxylase (TH, red) and MAP2 (green). Nuclei are counterstained with Hoechst. Scale bar, 50 µm. (I) Quantification of TH^+^ neurons as a percentage of total cells at day 50. Data are shown as mean ± SD. (J, K) Confocal images of TH^+^ neurons at day 50 showing punctate expression of synaptic markers SYN1 (green) and PSD95 (red). Scale bar, 20 µm. (L) Quantification of SYN1^+^ and PSD95^+^ puncta per 100 µm neurite length in TH^+^ neurons. Data are represented as mean ± SD from n = 20–30 cells per group.

The detailed sequences of the pegRNA and nicking gRNA used for prime editing are listed in Table S1. G‐banded karyotyping was performed on both the unedited parental hiPSCs and the gene‐corrected hiPSC lines (Figure S2A,B). No numerical or structural chromosomal aberrations, including translocations, deletions, duplications, or aneuploidies, were detected in either cell line. Both cell lines exhibited a normal karyotype with consistent banding patterns across all chromosomes, confirming that prime editing and subsequent clonal expansion did not exhibit genomic instability (Figure S2A,B). Sanger sequencing was employed on both the unedited and prime‐edited hiPSC lines. Sanger sequencing chromatograms from the edited hiPSCs displayed unambiguous single peaks at all examined genomic loci. (Figure S2C–F). No evidence of off‐target mutations or residual plasmid‐derived sequences was detected in the edited lines (Figure S2C–F). ELISA results showed that both L‐GC‐DA‐NPC (gene corrected group) and L‐Mut‐DA‐NPC (gene mutant group) exhibited dopamine release capacity, with no significant difference in dopamine release levels between these two groups, confirming that gene‐corrected neurons possess normal dopamine‐releasing capacity (Figure S2G).

### 2.2 Efficient Differentiation of iPSCs Into Midbrain Dopaminergic Progenitors and Neurons

A chemically defined, stage‐specific protocol was optimized to guide iPSC differentiation toward midbrain dopamine (DA) neuron lineage (Figure 1B). The protocol included early dual‐SMAD inhibition followed by SHH and CHIR99021‐mediated ventralization and subsequent exposure to maturation factors such as BDNF, GDNF, and cAMP. By day 16, neural progenitors exhibited robust expressions of LMX1, FOXA2, and NESTIN, consistent with a midbrain DA progenitor fate (Figure 1C).

Quantitative analysis of day‐16 cultures revealed high proportions of LMX1A^+^ and FOXA2^+^ cells. L‐Mut‐DA‐NPC exhibited 95.51% ± 0.96% LMX1A^+^ cells, 96.56% ± 1.11% FOXA2^+^ cells, and 93.53% ± 2.13% LMX1A^+^/FOXA2^+^ double‐positive cells, respectively. L‐GC‐DA‐NPC showed 93.61% ± 2.82% LMX1A^+^ cells, 94.57% ± 2.59% FOXA2^+^ cells, and 91.96% ± 0.83% LMX1A^+^/FOXA2^+^ double‐positive cells, respectively (Figure 1D,E,G). These findings were confirmed by both immunostaining and flow cytometry (Figure 1F). The co‐expression of these key markers across LRRK2 mutant and corrected lines was comparable, indicating uniform differentiation efficiency. At day 50, terminal differentiation yielded TH+ MAP2+ neurons, confirming successful acquisition of a dopaminergic neuronal identity (Figure 1H). Quantification of TH+ cells revealed equivalent differentiation capacity between L‐Mut‐DA‐NPC and L‐GC‐DA‐NPC lines with 83.30% ± 2.21% and 84.26% ± 2.21% of cells expressing TH, respectively (Figure 1I). Synaptic maturation was evident as TH+ neurons exhibited discrete punctate staining for the presynaptic marker SYN1 and postsynaptic density protein PSD95. Quantitative analysis of synaptic puncta densities revealed no significant differences between L‐Mut‐DA‐NPC and L‐GC‐DA‐NPC (6.50 ± 0.50 vs. 6.67 ± 0.47 puncta per 100 µm, respectively), supporting consistent synaptogenesis across genotypes (Figure 1J,K,L).

### 2.3 In Vivo Transplantation and Restoration of Dopamine Synthesis in MPTP‐Lesioned Primates

To test the therapeutic efficacy of the DA‐NPCs, we utilized an MPTP‐lesioned cynomolgus monkey model of PD [50, 51, 52]. MPTP was delivered using ALZET osmotic pumps over four weeks (0.1 mg/kg/day), followed by a 30‐day stabilization phase prior to cell transplantation (Figure 2A). Animals were randomly assigned to receive vehicle (PD group), L‐Mut‐DA‐NPCs, or L‐GC‐DA‐NPCs bilaterally into the striatum under MRI‐guided stereotactic surgery (Figures 2B and Figure S3A–C). Monkeys that received neither MPTP nor transplantation served as controls.

**FIGURE 2:**
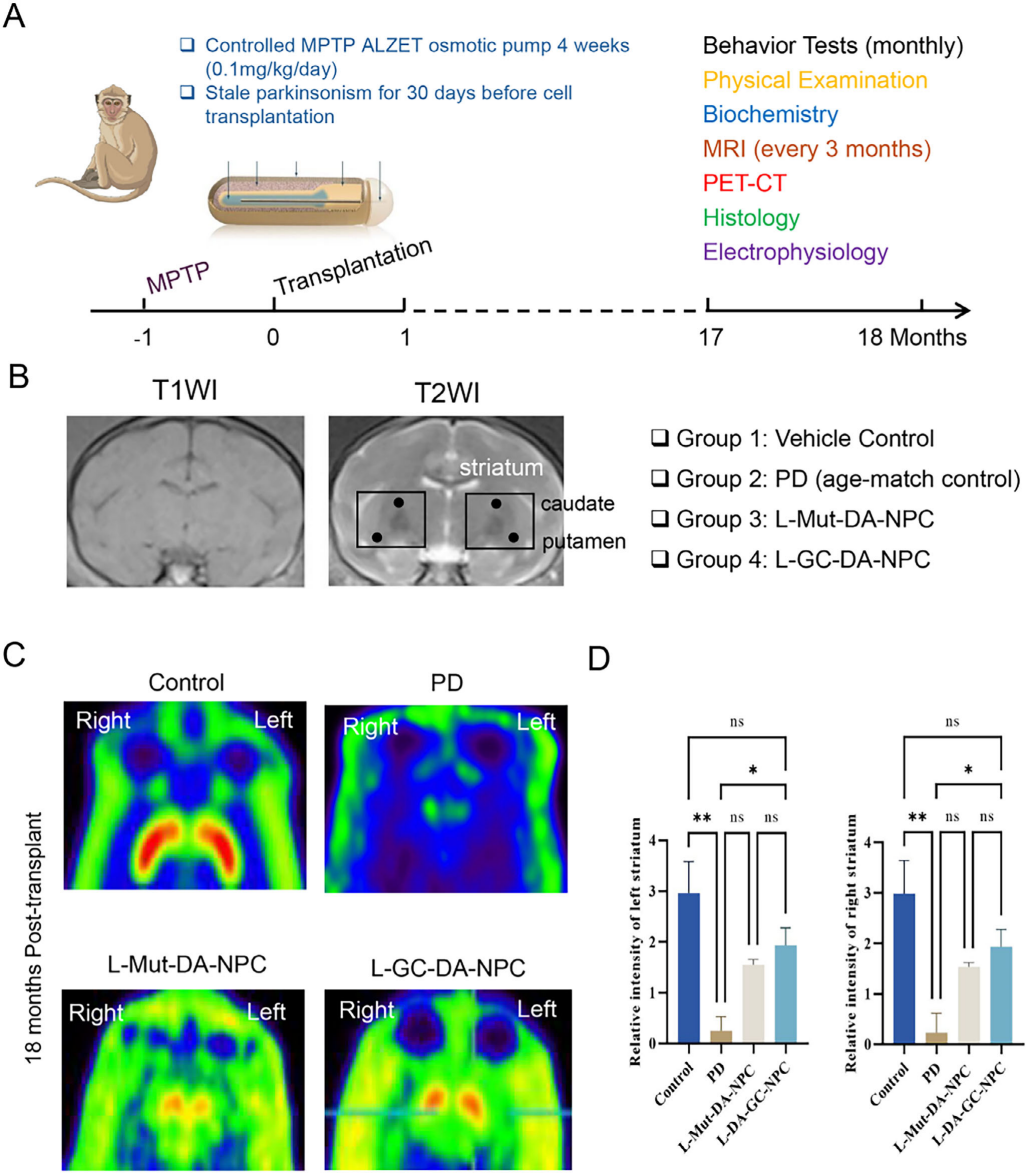
Transplanted dopaminergic progenitors restore dopamine synthesis in a primate PD model. (A) Schematic timeline of the experimental procedure. MPTP‐lesioned primates were stabilized for 30 days following a 4‐week infusion using an ALZET osmotic pump (0.1 mg/kg/day) before receiving cell transplantation. Longitudinal assessments included physical examination, biochemistry, MRI (every 3 months), PET‐CT, histology, and electrophysiology. (B) Representative MRI scans showing bilateral striatal graft sites targeted at dopaminergic cell transplantation. Insets highlight coronal and axial planes with striatal localization. (C) Representative [^1^ ^8^F] DOPA PET‐CT images at 18 months post‐transplantation, showing tracer uptake in the striata. L‐Mut‐DA‐NPC and L‐GC‐DA‐NPC transplanted groups exhibit enhanced radiotracer accumulation compared to the Parkinson's disease (PD) group. (D) Quantification of [^1^ ^8^F] DOPA uptake reveals significantly increased dopamine synthesis in the transplanted groups (L‐Mut‐DA‐NPC and L‐GC‐DA‐NPC) compared to the untreated PD group. Data are shown as mean ± SD, n = 2. ^*^ *p* < 0.05, ^**^ *p* < 0.01.

To assess shear stress effects during aspiration and dispensing, viability was evaluated by trypan blue exclusion before and after gentle aspiration into and injection from a Hamilton syringe. Syringe passage increased the dead cell fraction by only 1.1–2.1%, and overall viability remained >90% across all tested conditions, indicating that shear stress from Hamilton syringe handling is minimal and compatible with in vivo transplantation (Table S2).

To evaluate the safety of DA‐NPCs grafting, longitudinal MRI imaging, acquired at Pre‐MPTP, Pre‐grafting, and quarterly intervals up to 18 months post‐grafting, confirmed accurate, stable, and bilaterally symmetric graft placement within the caudate nucleus and putamen across the entire study duration. Longitudinal MRI imaging revealed that no abnormal mass formation, signal enhancement suggestive of proliferation, or signs of tumorigenicity were detected in any animal at any time point (Figure 2B and Figure S4).

To evaluate graft function, [^18^F] DOPA PET imaging [53, 54, 55, 56, 57] was performed at month 18 post‐transplantation. Transplanted animals exhibited robust recovery of [^18^F] DOPA uptake within the striatum (Figure 2C and Figure S5), and its quantitative data are shown in Table S3. Quantitative analysis of striatal [^1^ ^8^F]‐DOPA uptake revealed that the relative tracer intensities in both the left and right striatum were significantly higher in the L‐GC‐DA‐NPC and control groups than in the PD model group (Figure 2D). Although no statistically significant difference was observed between the L‐Mut‐DA‐NPC group and the PD model group (likely due to limited sample size and statistical power), there was also no significant difference between the L‐Mut‐DA‐NPC and L‐GC‐DA‐NPC groups. These results indicate that both mutant and gene‐corrected DA‐NPCs retain the capacity to restore dopamine synthesis in vivo. Bodyweight tracking revealed that while the PD group experienced progressive weight loss consistent with disease progression, both DA‐NPC groups maintained or recovered weight by 18 months post‐transplantation (Figure S3D).

### 2.4 Long‐Term Survival, Differentiation, and Integration of Grafted DA Neurons

To assess engraftment and cell fate *ex vivo*, we analyzed brain sections 18 months after transplantation. Whole‐mount 3D reconstruction of GFP+ cells revealed widespread distribution of graft‐derived cells throughout the striatal parenchyma (Figure 3A). High‐resolution imaging confirmed dense integration and long‐term survival of the grafted neurons (Figure 3B). Immunostaining demonstrated co‐expression of TH in GFP+ cells (Figure 3C), consistent with sustained dopaminergic identity. GIRK2, a marker of A9‐type substantia nigra DA neurons implicated in motor control [58, 59, 60], was also expressed in many GFP+ cells (Figure 3D), indicating subtype‐specific differentiation. Quantitative analysis showed that the proportions of GFP+ cells co‐expressing TH and GIRK2 were comparable between L‐Mut‐DA‐NPC and L‐GC‐DA‐NPC groups. Specifically, TH^+^/GFP^+^ cells were 95.87% ± 2.94% (L‐Mut‐DA‐NPC) versus 97.60% ± 2.45% (L‐GC‐DA‐NPC), and GIRK2^+^/GFP^+^ cells were 96.84% ± 2.36% versus 95.63% ± 2.58%, respectively (Figure 3E,F), suggesting gene correction did not impact in vivo neuronal identity or maturation.

**FIGURE 3:**
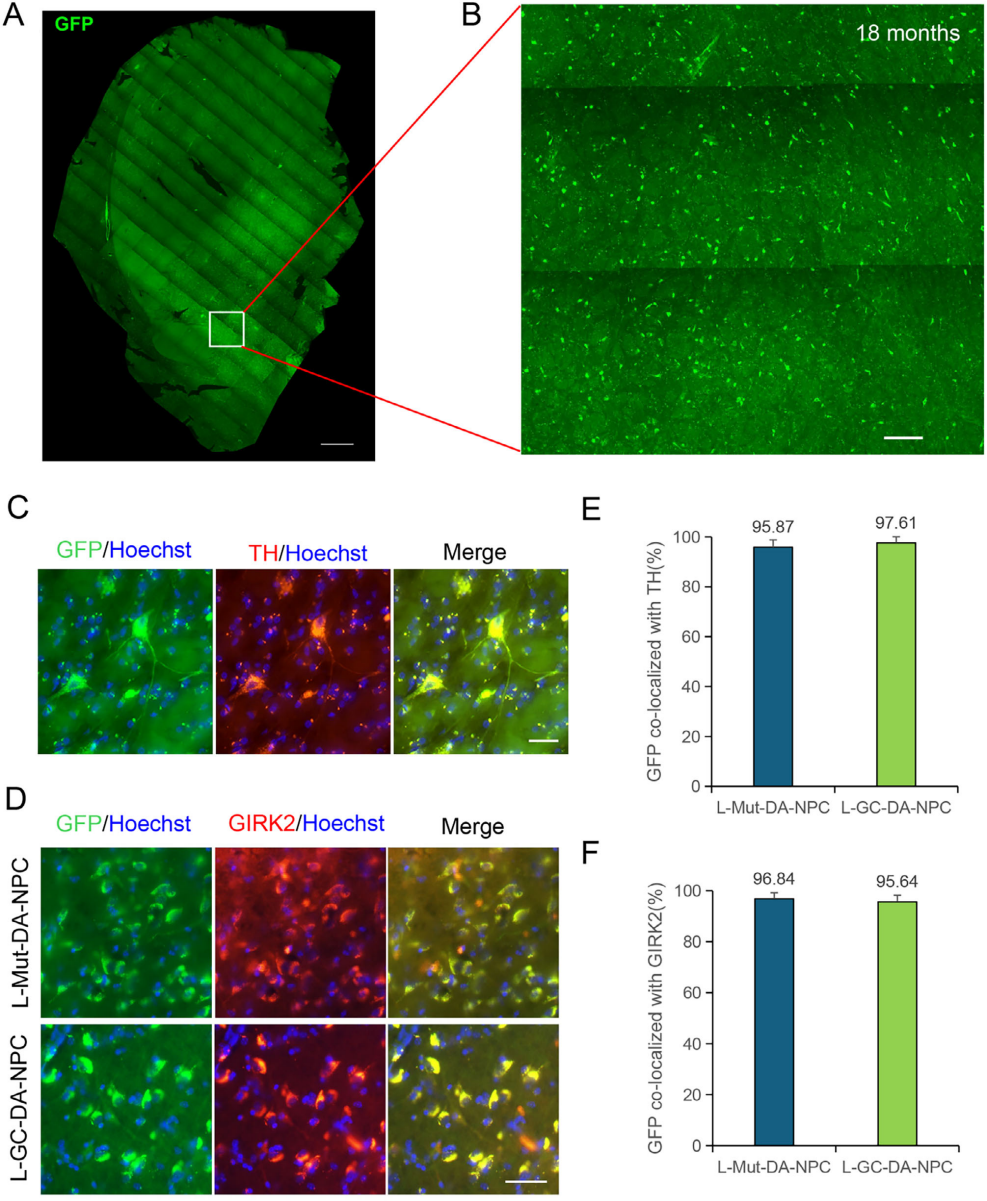
Long‐term survival, distribution, and dopaminergic phenotype of transplanted iPSC‐derived DA progenitors in the striatum of MPTP‐lesioned primates. (A) Low‐magnification 3D reconstruction showing broad distribution of GFP^+^ transplanted cells within the primate striatum at 18 months post‐transplantation. Scale bar, 1.6 mm. (B) Higher magnification view of the boxed region in (A), illustrating dense integration of GFP^+^ cells throughout the striatal parenchyma. Scale bar, 100 µm. (C) Representative immunofluorescence images showing GFP^+^ cells (green) co‐expressing tyrosine hydroxylase (TH, red), indicating dopaminergic identity. Nuclei are counterstained with Hoechst (blue). Scale bar, 50 µm. (D) Immunofluorescence staining for GIRK2 (red), a marker of A9‐type dopaminergic neurons, in GFP^+^ transplanted cells. Hoechst marks nuclei (blue). Scale bar, 50 µm. (E,F) Quantification of the percentage of GFP^+^ cells co‐expressing human TH (E) and GIRK2 (F). No significant differences were observed between the LRRK2‐mutant (L‐Mut‐DA‐NPC) and isogenic corrected (L‐GC‐DA‐NPC) grafts. Data is presented as mean ± SD.

At the 18‐month endpoint, robust GFP^+^ grafts were detected in the striatum (Figure 3A,B), with more than 95% surviving cells co‐localizing with the dopaminergic markers TH (Figure 3C,E) and GIRK2 (Figure 3D,F). Quantitative analysis confirmed no significant difference in the proportion of TH^+^ and GIRK2^+^ cells between the L‐Mut‐DA‐NPC and L‐GC‐DA‐NPC groups, demonstrating that gene correction did not impair the survival or differentiation potential of the grafted cells.

### 2.5 Minimal Astrocytic Response to DA‐NPC Grafts

Host immune response to the graft was evaluated via immunohistochemistry for glial fibrillary acidic protein (GFAP), a marker of reactive astrocytes [61, 62]. Confocal imaging revealed limited GFAP expression surrounding the graft in all groups (Figure S6A). Quantitative fluorescence intensity measurements showed no significant increase in astrocytic reactivity in either DA‐NPC group compared to controls (L‐Mut‐DA‐NPC: 1.03 ± 0.05; L‐GC‐DA‐NPC: 1.01 ± 0.04; Control: 1.00 ± 0.03, normalized as fold change relative to control) (Figure S6B), suggesting that both LRRK2 mutant and gene‐corrected grafts were well tolerated without eliciting chronic glial activation.

### 2.6 Grafted DA Neurons Exhibit Mature Electrophysiological Properties

To test the electrophysiological maturity and functionality of the grafted neurons, we performed whole‐cell patch‐clamp recordings. Briefly, the brain tissue containing the putamen was immediately collected from euthanized animals and sectioned into 300‐um slices. The slices were maintained in artificial cerebrospinal fluid (ACSF) and continuously oxygenated until recording. Under a fluorescent microscope, grafted neurons (18 months post‐grafting) labeled with GFP^+^ antibody were identified and examined. These neurons exhibited typical dopaminergic neuronal firing properties. Specifically, they generated spontaneous action potentials (APs) at the resting membrane potential and fired repetitively in response to depolarizing current injection (Figure 4A–C). Both responses were abolished by tetrodotoxin (TTX) application, confirming functional sodium channel activity in the grafted neurons (Figure 4D). Moreover, these grafted DA neurons displayed postsynaptic currents (Figure 4E), indicating that they received the synaptic inputs from host neurons and had been integrated into the host neuronal networks.

**FIGURE 4:**
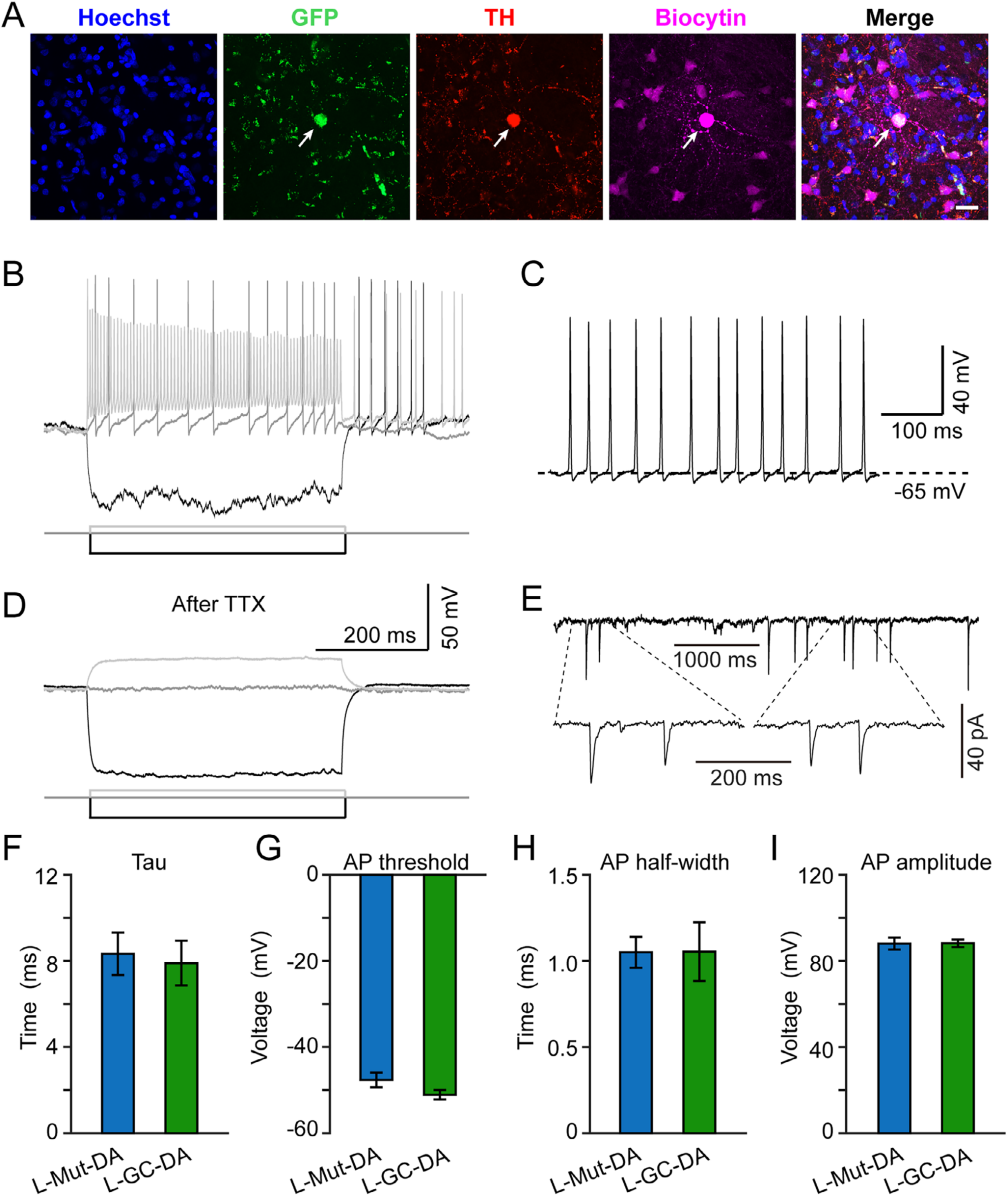
Functional maturation and electrophysiological properties of grafted human midbrain dopamine neurons at 18 months post‐transplantation. (A) Representative immunofluorescence images of a grafted GFP^+^ cell co‐expressing tyrosine hydroxylase (TH, red) and filled with biocytin (magenta) to visualize neuronal morphology. Nuclei are counterstained with Hoechst (blue). The white arrows indicate the recorded cell. Scale bar, 50 µm. (B) Firing pattern of an example grafted human dopamine neuron in response to injected currents (bottom). Black: hyperpolarization trace; dark gray: nearing threshold trace; bright gray: depolarization trace. Injection currents: −180, 0, and 80 pA. (C) spontaneous activities of the same neuron shown in B. The dashed line shows the resting membrane potential (−65 mV). (D) Similar to B, voltage response of the same neuron after application of tetrodotoxin (TTX), revealing blockade of action potential firing, consistent with sodium channel dependence. The same injection currents as those in B were depicted at the bottom. (E) Example trace showing spontaneous excitatory postsynaptic currents (EPSCs) recorded from a grafted neuron. Bottom magnifications showed example EPSC events. (F–I) Quantification of intrinsic membrane properties of grafted neurons derived from LRRK2‐mutant (L‐Mut‐DA‐NPC) and gene‐corrected (L‐GC‐DA‐NPC) iPSC lines. Measured parameters include: (F) membrane time constant (tau); (G) AP threshold; (H) AP half‐width; (I) AP amplitude. No significant differences were observed between groups (the p values were 1, 0.44, 0.61, and 1.0, respectively, after Bonferroni correction for (F–I). Data are presented as mean ± SEM (n = 27 for L‐Mut_DA and n = 55 for L‐GC‐DA, respectively).

The electrophysiological properties were extracted and digitized. The main intrinsic properties, including membrane time constant (tau), AP amplitude, AP half‐width, and AP threshold, were comparable between L‐Mut and L‐GC grafts (Figure 4F–I). A detailed summary of membrane and action potential characteristics is provided in Table S4. These results demonstrate that both mutant and corrected DA‐NPCs give rise to electrophysiologically mature neurons capable of integrating into host neural circuitry.

### 2.7 Behavioral Recovery in DA‐NPC‐Transplanted Primates

As shown in Table S5, Hoehn‐Yahr (H&Y) scores remained stable in all MPTP‐lesioned non‐human primates from 30 days post‐lesion induction through the 18‐month follow‐up period, confirming the absence of spontaneous functional recovery and validating the chronicity of the model.

Behavioral recovery was assessed by open‐field testing at multiple time points over 18 months. Heatmap‐based spatial trajectory analysis revealed severely restricted and stereotyped movements in the PD model group. In contrast, both DA‐NPC‐transplanted groups showed markedly enhanced locomotor activity and exploratory behavior (Figure 5B). The L‐GC‐DA‐NPC group displayed the most consistent and extensive recovery, as measured by quadrant‐specific activity metrics.

**FIGURE 5:**
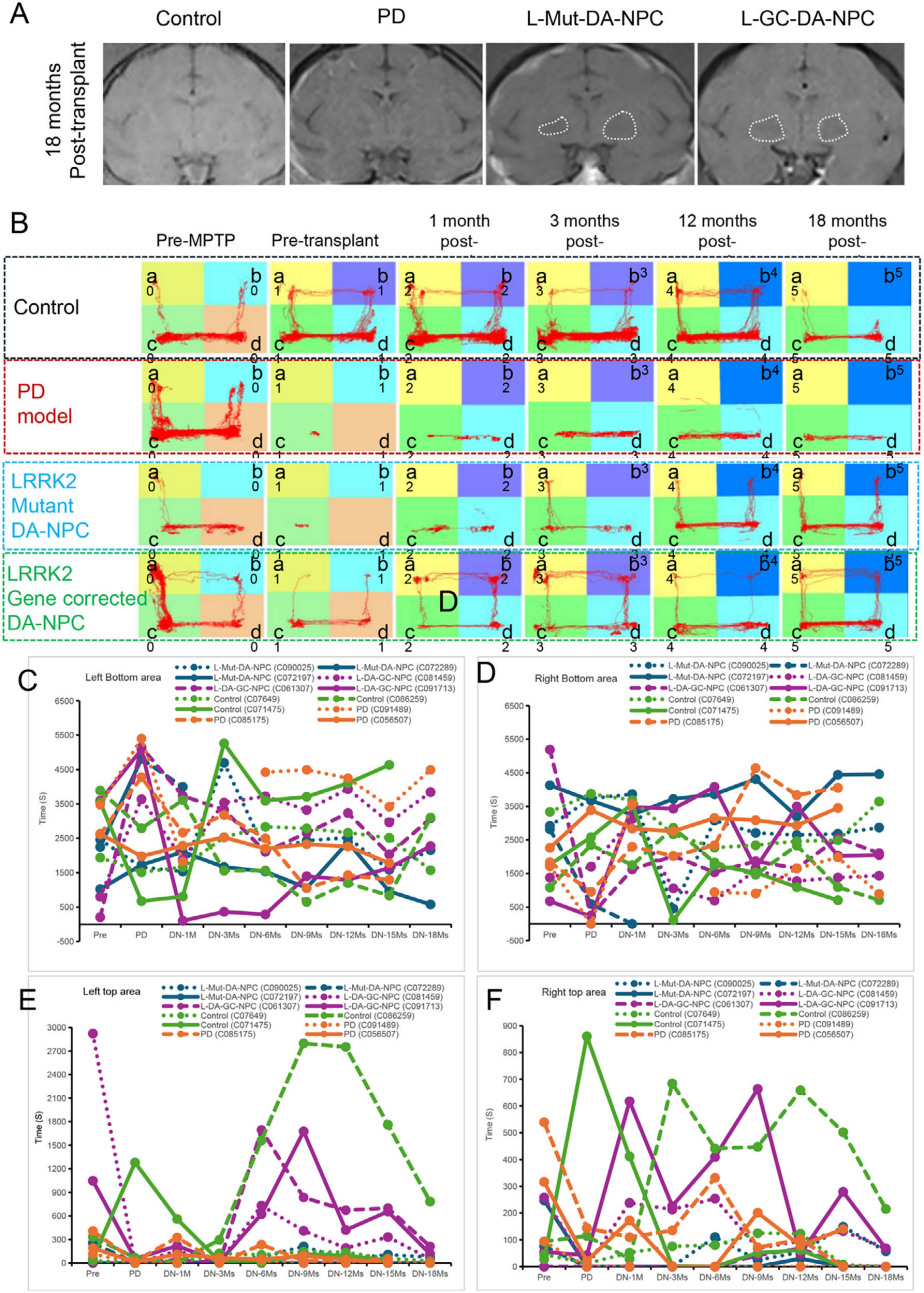
Behavioral tracking reveals motor improvements following DA‐NPC transplantation in MPTP‐lesioned primates. (A) Safety assessment of long‐term DA‐NPC grafts reveals no tumorigenicity at 18 months post‐transplantation. Representative magnetic resonance imaging (MRI) scans from monkeys at 18 months post‐transplantation. Coronal and axial sections of the striatum show no evidence of tumor formation or abnormal mass at the graft site. (B) Heatmap‐based locomotor trajectory analysis of individual monkeys during open‐field testing. Each row represents an individual animal from the following groups: Top row (red box): MPTP‐induced Parkinson's disease (PD) model without treatment. Middle row (blue box): LRRK2‐mutant DA‐NPC transplantation group. Bottom row (green box): Isogenic gene‐corrected DA‐NPC transplantation group. Each panel (a–d, a'–d', etc.) corresponds to spatial trajectories recorded in defined grid zones across sequential time points. PD model animals exhibit reduced and restricted locomotor activity, while transplanted groups, especially gene‐corrected DA‐NPC recipients, show enhanced exploration and restored motor behaviors over time. These findings suggest long‐term functional integration of transplanted neurons and partial recovery of motor function. (C) Locomotor activity in the left bottom quadrant. Pre‐MPTP & Pre‐transplant: High baseline locomotor activity is observed across nearly all animals, especially controls and L‐GC‐DA‐NPC recipients, indicating healthy motor function prior to lesioning. Post‐MPTP (1–3 Months): PD model animals exhibit sharp declines in movement, a hallmark of successful dopaminergic lesion. L‐Mut‐DA‐NPC animals show variable, partial recovery. L‐GC‐DA‐NPC animals demonstrate a more consistent and progressive return of movement, closely resembling or surpassing controls by 18 months. 18 Months: The L‐GC‐DA‐NPC group maintains high locomotor activity, consistent with effective functional integration. The PD group shows no recovery, while L‐Mut‐DA‐NPC remains heterogeneous. (D) Locomotor activity in the right bottom quadrant at 18 months post‐transplantation. Bar graph showing quantification of spontaneous locomotor activity in the right bottom quadrant of the open‐field arena at 18 months following transplantation. Activity was measured as cumulative movement time (arbitrary units) and averaged for each experimental group: Control: Animals without MPTP lesion or cell transplantation. PD model animals: MPTP‐lesioned animals without transplantation. L‐Mut‐DA‐NPC: MPTP‐lesioned animals receiving LRRK2‐mutant dopaminergic progenitors. L‐GC‐DA‐NPC: MPTP‐lesioned animals receiving isogenic gene‐corrected dopaminergic progenitors. LRRK2 mutant graft recipients exhibited elevated activity with greater variability, while gene‐corrected recipients showed moderate and consistent recovery. PD‐model animals exhibited minimal movement, confirming sustained motor impairment. Data is shown as mean ± SD. (E) Locomotor activity in the left top quadrant. Pre‐MPTP: All animals show relatively low and comparable movement. Pre‐transplant: Activity drops substantially in most groups, especially the PD and mutant DA‐NPC animals, confirming effective MPTP lesioning. 1–18 Months: L‐GC‐DA‐NPC animals show progressive and sustained recovery over time. L‐Mut‐DA‐NPC animals show partial recovery with variability between individuals. PD model animals display sustained or erratic elevation, possibly reflecting dyskinesia or behavioral compensation. Control animals maintain consistent, lower‐level activity throughout, indicating baseline movement without MPTP effect. (F) Locomotor activity in the right top quadrant at 18 months post‐transplantation. Line plot showing time‐series locomotor activity of individual animals in the right top quadrant of the open‐field test from pre‐MPTP through 18 months post‐transplantation. Movement is quantified as time spent actively exploring within the region. Across all groups: PD‐model animals exhibited persistently reduced movement, indicating sustained motor dysfunction. L‐Mut‐DA‐NPC animals showed partial recovery with some individual variation. L‐GC‐DA‐NPC animals showed modest but consistent activity, supporting functional graft integration. Control animals retained baseline low movement levels typical of non‐lesioned exploratory behavior. At 18 months, differences between groups were subtle in the right top quadrant compared to the lower quadrants, suggesting region‐specific behavioral engagement or motor asymmetry. Raw individual quantitative data underlying Figure 5C–F are provided in Table S6, and detailed definitions of missing values and zero value measurements are specified within the legend of Table S6. Lines in Figure 5C–F are omitted between discrete time points where behavioral monitoring was not performed.

Quantitative analysis across quadrants showed that L‐GC‐DA‐NPC recipients had significantly higher movement in the left bottom and right bottom quadrants, and moderate but sustained activity in the left top and right top quadrants (Figure 5C–F andTable S6). The L‐Mut‐DA‐NPC group exhibited partial behavioral recovery with individual variability (Figure 5C–F and Table S6). PD animals remained hypoactive throughout the testing period (Figure 5C–F and Table S6). These findings indicate that DA‐NPC grafts improved motor function in MPTP‐lesioned primates, with gene‐corrected cells conferring the most consistent benefit.

### 2.8 Long‐Term Safety of DA‐NPC Grafts

Serum chemistry analyses for tumorigenicity and systemic toxicity revealed no significant changes in AFP, CEA, or NSE (Figure 6A–C and Table S7). Liver function tests (ALT, AST), renal biomarkers (BUN, CRE), and LDH were retained within physiological ranges across all time points and treatment groups (Figure 6D‐H  and Table S7). Serum liver function tests (ALT, AST) revealed distinct group differences. The PD model group exhibited a progressive and significant elevation in ALT and AST levels over 18 months, consistent with MPTP‐induced chronic neurodegeneration and secondary systemic oxidative stress. In contrast, both the L‐Mut‐DA‐NPC group and L‐GC‐DA‐NPC group maintained ALT and AST levels within normal physiological ranges and no statistically significant inter‐group difference throughout the observation period, confirming no hepatotoxicity associated with DA‐NPC transplantation (Figure 6D–E). To assess potential tumorigenicity or abnormal proliferation, all animals underwent longitudinal MRI examinations at Pre‐MPTP baseline, pre‐grafting, and every 3 months post‐grafting (3, 6, 9, 12, 15, and 18 months). No abnormal mass formation, signal enhancement suggestive of proliferation, or signs of tumorigenicity were detected in any animal at any time point (Figures 5A and S4). These results support the long‐term safety, tolerability, and non‐tumorigenic nature of both L‐Mut and L‐GC DA‐NPC grafts.

**FIGURE 6:**
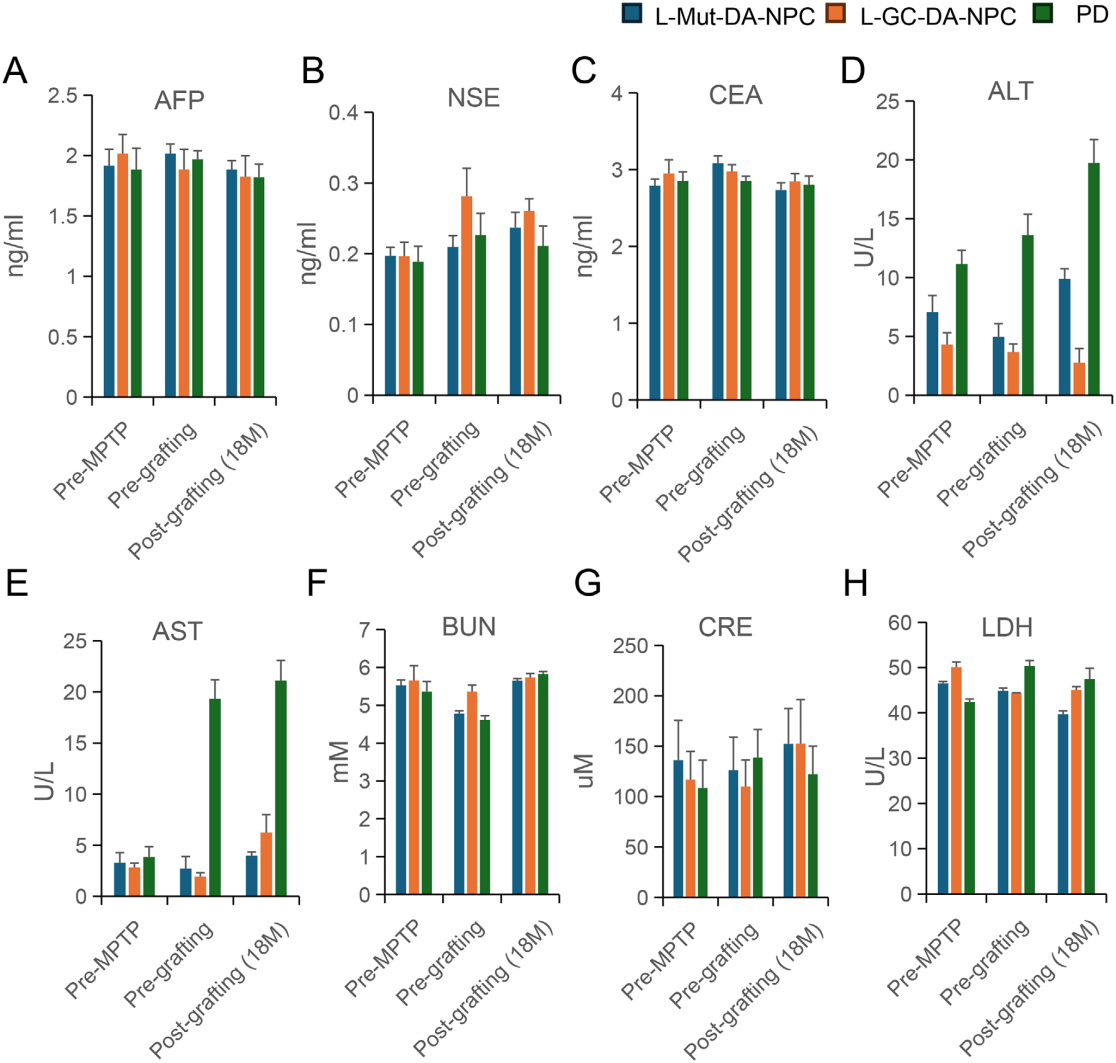
Systemic safety assessment in MPTP‐lesioned monkeys. Systemic safety profile assessed through serum biochemistry and tumor marker assays at three time points: pre‐MPTP lesion, pre‐grafting, and 18 months post‐transplantation. No significant abnormalities or upward trends were observed across time points in the following markers: (A) AFP (α‐fetoprotein) – liver tumor marker. (B) NSE (neuron‐specific enolase) – neuroendocrine tumor marker. (C) CEA (carcinoembryonic antigen) – gastrointestinal tumor marker. (D, E) ALT/AST – liver function and hepatotoxicity indicators. (F, G) BUN/CRE – renal function. H) LDH – tissue damage and cell turnover marker.

## 3 Discussion

In this study, we demonstrate the long‐term safety, integration, and functional efficacy of human iPSC‐derived midbrain dopaminergic progenitors (DA‐NPCs) transplanted into a non‐human primate model of Parkinson's disease (PD). Using isogenic iPSC lines harboring and corrected for pathogenic LRRK2 mutations, we show that both mutant and gene‐corrected DA‐NPCs are capable of robust in vitro differentiation, sustained in vivo survival, and significant behavioral rescue following transplantation into MPTP‐lesioned monkeys. Importantly, our findings indicate that gene correction does not impair the capacity of DA‐NPCs to engraft, mature, and restore dopaminergic function, and that long‐term grafts do not exhibit tumorigenicity or adverse immune responses.

We first established a PD iPSC model based on compound LRRK2 mutations (R50H and M2397T) and validated pluripotency and trilineage potential across both mutant and gene‐corrected lines. Directed differentiation yielded DA‐NPCs expressing key midbrain markers (LMX1, FOXA2, NESTIN) and terminally differentiated TH+ MAP2+ neurons with synaptic features. These data confirm that disease‐associated mutations did not preclude effective DA lineage commitment or terminal differentiation. The choice of a patient with compound heterozygous LRRK2 mutations warrants consideration. While autosomal dominant single heterozygous LRRK2 mutations are more common in familial PD, compound heterozygous genotypes, although rare, can lead to early‐onset or more severe clinical presentations [63, 64]. The patient in this study was selected based on sample availability and thorough prior genetic characterization. Importantly, our study was not designed to dissect the individual contribution of each mutation; rather, it aimed to determine whether gene correction of the patient‐specific pathogenic variants restores functional competence and safety of autologous iPSC‐derived dopaminergic progenitors in a clinically relevant non‐human primate model. The isogenic experimental design, comparing uncorrected and fully corrected lines derived from the same donor, eliminates confounding effects attributable to genetic background variation and enables direct assessment of the functional consequences of mutation correction.

In this current study, prime editor plasmids were utilized to establish gene‐corrected hiPSC lines for cell transplantation research. Although routine Sanger sequencing confirmed precise on‐target editing and excluded obvious exogenous sequence insertion at the editing loci, specific quantitative detection of residual plasmid fragments in edited cells was not performed in this work. Episomal plasmids are typically diluted and eliminated during extended passaging of human pluripotent stem cells, and the prime editing system exhibits minimal intrinsic risk of random genomic integration under standardized culture conditions [34, 65]. However, residual exogenous DNA remains a critical safety consideration for clinical translation. To rigorously assess plasmid clearance, subsequent studies will implement highly sensitive, quantitative PCR‐based assays to systematically quantify residual plasmid DNA. These efforts will further enhance the safety and translational readiness of gene‐edited, hiPSC‐derived neuronal progenitors. Notably, this study comprehensively evaluated iPSC pluripotency, neuronal differentiation efficiency, and synaptic maturation in vivo; additionally, genomic stability and dopamine release were rigorously assessed following protocol refinement. However, in vitro analyses of canonical Parkinson's disease‐associated phenotypes, including mitochondrial dysfunction and electrophysiological deficits, were not included. As the primary objective was to establish the in vivo therapeutic efficacy of gene‐edited cells, mechanistic investigations of cell‐autonomous PD pathophysiology will be carried out in subsequent studies.

In this study, we established a chronic Parkinson's disease model in aged cynomolgus monkeys (～13 years old, equivalent to elderly humans) using continuous subcutaneous infusion of MPTP via ALZET osmotic minipumps. We acknowledge that prolonged post‐MPTP stabilization periods have been employed in certain non‐human primate PD models, particularly in younger vervet monkeys, where spontaneous behavioral recovery has been documented [66]. In contrast, our aged non‐human primate model, established via chronic, low‐dose MPTP infusion using ALZET osmotic minipumps, exhibits enhanced clinical relevance and longitudinal stability, faithfully recapitulating the progressive neurodegeneration characteristic of human Parkinson's disease. Critically, this approach minimizes spontaneous functional recovery and yields highly consistent dopaminergic neuron loss. The H&Y scores remained stable for at least 30 days following intoxication, and longitudinal behavioral assessments of monkeys in the PD group confirmed persistent motor deficits from model induction through 18 months post‐transplantation. This exceptional temporal stability renders the model highly suitable for rigorous evaluation of cell therapy efficacy, effectively mitigating confounding effects attributable to spontaneous recovery and thereby strengthening the validity and robustness of our conclusions.

Although conventional perspectives have held that MPTP‐based non‐human primate models of Parkinson's disease fail to fully recapitulate the progressive and multifactorial neuropathology of the human condition, particularly with respect to non‐dopaminergic involvement and widespread neurodegeneration [67], recent evidence indicates that this limitation is largely confined to acute MPTP administration protocols [68]. As demonstrated by Masilamoni and Smith, chronic, low‐dose MPTP exposure in non‐human primates induces a broad pathological profile extending beyond nigrostriatal dopaminergic degeneration to include neuronal loss in non‐dopaminergic populations across the brainstem, thalamus, and other extrastriatal regions [63]. This pattern closely mirrors the mixed neuropathological landscape characteristic of human Parkinson's disease. Consistent with these findings, the chronic, low‐dose MPTP regimen delivered via ALZET osmotic minipumps in this current study was specifically designed to model this expanded spectrum of neurodegeneration. Consequently, our model exhibits enhanced clinical fidelity compared with acute toxin‐based paradigms and is well‐suited for evaluating both motor and non‐motor functional outcomes following cell transplantation therapy.

Transplantation into MPTP‐lesioned cynomolgus monkeys resulted in long‐term graft survival and broad striatal integration. PET imaging at 18 months showed enhanced [^18^F] DOPA uptake in transplanted groups, indicating restored dopamine synthesis. Quantitatively, both L‐Mut‐DA‐NPC and L‐GC‐DA‐NPC recipients exhibited significantly elevated tracer uptake compared to the untreated PD group, with no statistically significant difference between these two groups. Given the small sample size, this current study is underpowered to detect subtle functional differences between groups. Hemispheric variability in [^18^F] DOPA uptake was observed across animals, which may reflect differences in graft placement, local microenvironment variations, or intrinsic biological variability. Importantly, such hemispheric asymmetry of the dopaminergic system is a fundamental, evolutionarily conserved neurophysiological feature in primates, including humans, rather than a pathological deviation or anomaly [69]. Notably, we did not observe a consistent correlation between hemispheric PET asymmetry and post‐mortem electrophysiological or histological endpoints. For clinical translation, high cell survival and maintenance of dopaminergic identity are critical factors for functional efficacy and safety.

An important motivation for the present study was to address the ongoing debate regarding the necessity of gene correction in autologous iPSC‐based therapies for genetic PD. While it has been argued that disease‐related phenotypes may take decades to manifest in transplanted cells, and thus correction may not be essential for short‐ to medium‐term graft function [70], our empirical data in a primate model provide key insights into this question. Critically, LRRK2‐mutant and gene‐corrected DA‐NPCs exhibited comparable in vivo performance throughout the 18‐month study period, with no statistically significant differences in graft survival, expression of dopaminergic markers, electrophysiological maturation, or dopamine synthesis capacity. These findings demonstrate that autologous iPSC‐derived DA‐NPCs generated from an LRRK2‐linked Parkinson's disease patient support safe and functionally competent engraftment in the absence of prior gene correction. This supports the therapeutic feasibility of autologous iPSC‐based approaches for genetically defined forms of Parkinson's disease.

Importantly, both mutant and corrected DA‐NPCs survived and matured in vivo, with high proportions of graft‐derived cells co‐expressing TH and GIRK2, a marker of A9 DA neuron subtype relevant to motor function, with >95% of surviving GFP^+^ cells co‐expressing TH and GIRK2 at the 18‐month endpoint. These data provide important reassurance that both the mutant and gene‐corrected cell products exhibit robust survival and lineage stability in the primate brain, supporting their potential as safe and effective candidates for cell replacement therapy. Moreover, while TH^+^/GFP^+^ cells were readily detected in the striatum, precise determination of absolute cell survival efficiency was not possible due to the use of representative sectional analysis rather than comprehensive three‐dimensional reconstruction, which restricts full quantification of the total surviving cell population. However, Electrophysiological recordings confirmed functional maturation, as evidenced by spontaneous and evoked action potentials, as well as synaptic activity in the grafted cells.

Histological analysis focused on the striatum, the primary transplantation site, and the anatomical region most critically involved in dopaminergic circuit reconstruction in Parkinson's disease [71]. Consistent with our neuropathological findings, GFP‐positive grafted cells were predominantly localized within the striatal parenchyma and exhibited high‐density engraftment. Critically, these cells displayed mature neuronal morphology and robust expression of dopaminergic markers, confirming their identity as post‐mitotic, functionally committed dopaminergic neurons, not residual progenitors. Mature neurons derived from pluripotent stem cells exhibit markedly reduced migratory capacity relative to their progenitor counterparts, thereby restricting engraftment largely to the injection site. Although we cannot exclude the possibility of minimal, non‐functional dispersion beyond the striatum, comprehensive histological examination across multiple brain regions revealed no evidence of substantial off‐target cell accumulation.

Several recent long‐term studies have demonstrated the feasibility of dopaminergic neuron replacement using human iPSC‐derived or pluripotent stem cell‐derived grafts in non‐human primate models of Parkinson's disease, reporting safe engraftment, striatal reinnervation, and sustained behavioral improvement over extended follow‐up periods [43, 46, 47, 48, 49]. Consistent with these findings, this current study confirms robust long‐term survival, functional maturation, and progressive behavioral recovery following transplantation of human iPSC‐derived DA‐NPCs. Crucially, whereas prior studies predominantly utilized wild‐type or allogeneic donor cells, this study directly compares isogenic LRRK2‐mutant and gene‐corrected autologous DA‐NPCs within an identical genetic background, thereby enabling a rigorously controlled assessment of how *LRRK2* gene correction influences graft survival, maturation, and functional integration in a clinically relevant, chronic non‐human primate model.

Notably, behavioral analysis revealed significant and sustained locomotor recovery in transplanted animals. Gene‐corrected DA‐NPCs conferred more consistent motor improvements, while LRRK2‐mutant grafts yielded variable outcomes across individual animals. This variability may reflect subtle differences in graft‐host integration or intrinsic resilience of corrected cells, despite equivalent TH+ and GIRK2+ profiles. Given that [^18^F] DOPA uptake was only modestly higher in the L‐GC‐DA‐NPC group, it is unlikely that dopamine synthesis alone fully accounts for the behavioral differences observed. Rather, improved cell survival, synaptic connectivity, or enhanced integration into motivational and limbic circuitry may contribute to the superior functional outcomes in the gene‐corrected group. Notably, motor symmetry, a clinically validated parameter for Parkinson's disease assessment, was not formally evaluated in this study; our behavioral assessment focused on overall locomotor activity and exploratory behavior. Incorporating quantitative assessment of interlimb coordination and bilateral motor balance represents a key opportunity to enhance the granularity and clinical translatability of functional outcome measures in future investigations.

Interestingly, beyond improvements in ground‐level locomotion, we also observed enhanced activity in the upper regions of the test enclosure, specifically, the left top and right top quadrants corresponding to elevated ceiling structures such as perches or climbing zones. Increased engagement with these vertical spaces in transplanted animals, particularly in L‐GC‐DA‐NPC recipients, suggests restoration of goal‐directed exploratory behavior. Such vertical exploration, typically diminished in MPTP‐lesioned primates, may reflect alleviation of non‐motor symptoms commonly associated with PD, including apathy, anxiety, and reduced environmental engagement. In line with this, a previous study by Tao et al., also reported reduced depressive‐like behaviors in MPTP‐lesioned non‐human primates following stem cell‐based therapy for Parkinson's disease [49]. These findings raise the possibility that DA‐NPC transplantation confers not only motor improvements, but also partial recovery of affective and motivational functions mediated by dopaminergic and limbic circuits.

Our study also provides strong evidence for the safety of long‐term DA‐NPC grafts. MRI and histology showed no signs of tumorigenesis, and systemic blood markers retained within physiological ranges across all animals. Minimal astrocytic response was observed near graft sites, consistent with the low immunogenicity of human DA‐NPCs in this xenograft model.

Together, these results support the therapeutic potential of human iPSC‐derived DA‐NPCs for PD and validate a clinically relevant, long‐term primate model for translational development. Importantly, the comparable in vivo performance of LRRK2‐mutant and gene‐corrected DA‐NPCs suggests that patient‐derived autologous iPSC therapies may be feasible, even in the context of genetic forms of PD. Future studies are warranted to evaluate immune compatibility in allogeneic settings, optimize cell dose and delivery strategies, and determine the durability of clinical benefit in longer timeframes. Our findings provide compelling preclinical evidence that human iPSC‐derived midbrain dopaminergic progenitors can safely and effectively integrate into the primate brain, restore dopamine synthesis, and improve both motor and non‐motor behaviors associated with Parkinson's disease. By leveraging isogenic LRRK2‐mutant and gene‐corrected iPSC lines, we also demonstrate that gene correction enhances the therapeutic outcomes without compromising differentiation potential or graft viability. These results reinforce the feasibility of personalized, genetically tailored cell therapy for PD and underscore the critical importance of long‐term, multidimensional assessment in clinically relevant animal models. Nevertheless, our findings carry significant implications for advancing personalized cell therapy in PD. The demonstration that gene‐corrected autologous iPSC‐derived DA progenitors perform comparably, indicating that autologous iPSC‐based therapy is feasible even for patients with genetic Parkinson's disease. Our approach underscores the feasibility of combining precision modeling with therapeutic development in a single iPSC platform, paving the way for individualized treatment paradigms in neurodegenerative disease.

Several limitations must be acknowledged, while our study establishes a strong preclinical foundation for iPSC‐based DA neuron replacement therapy. First, the small sample size and inherent variability in primate studies limit the statistical power to detect subtle differences, particularly between gene‐corrected and mutant graft outcomes. Accordingly, no claims regarding the superior efficacy of gene‐corrected cells can be made based on the present data. Second, while our behavioral analyses include multidimensional assessments, they remain observational and semi‐quantitative; future studies incorporating automated scoring systems and more specific non‐motor testing will be important. Moreover, although our data suggests a trend toward enhanced dopamine synthesis in gene‐corrected grafts, the absence of significant differences in TH or GIRK2 expression complicates mechanistic interpretation. Third, the donor harbors a compound heterozygous LRRK2 genotype (R50H/M2397T), a rare configuration in Parkinson's disease. As this study was not designed for allele‐specific interrogation, the individual contributions of each variant to cellular phenotypes or therapeutic outcomes remain unresolved, limiting direct extrapolation to the more prevalent heterozygous LRRK2‐associated PD. Fourth, immunological considerations warrant further investigation. Although minimal astrocytic reactivity and no evidence of tumorigenicity were observed, the xenogeneic transplantation setting and concomitant immunosuppression may obscure clinically relevant host‐graft interactions. Fifth, the long‐term influence of LRRK2 mutations on graft stability, α‐synuclein pathology, and host circuit remodeling remains undefined. Sixth, histological analysis was restricted to the striatum; systematic evaluation of cell distribution across other brain regions was not performed. While mature dopaminergic neurons exhibit inherently limited migratory capacity, low‐level off‐target dispersion cannot be excluded, representing a methodological constraint of this study. Seventh, although GIRK2 expression confirmed the presence of A9‐type dopaminergic neurons, critical for motor recovery, calbindin staining was not conducted to definitively distinguish A9 from A10 subtypes, thus limiting comprehensive characterization of graft composition. Finally, translation to human trials will require stringent Good Manufacturing Practice (GMP)‐grade derivation protocols, reproducibility across diverse patient backgrounds, and a better understanding of optimal delivery parameters and dosing.

## 4 Conclusion

In summary, we establish a stable, chronic Parkinson's disease model in aged non‐human primates that faithfully recapitulates the progressive neuropathology of Human PD. Using isogenic LRRK2‐mutant and gene‐corrected human iPSC lines, we demonstrate that autologous dopaminergic neural progenitor cells, regardless of LRRK2 status, exhibit robust neuronal differentiation, long‐term graft survival, extensive striatal reinnervation, and outstanding safety. Sustained dopaminergic restoration drove significant, durable improvements in motor deficits. Critically, LRRK2‐mutant cells performed equivalently to corrected isogenic controls over 18 months post‐transplantation, providing direct preclinical evidence that uncorrected autologous iPSC‐derived neurons can engraft safely and functionally in genetically defined PD. These findings deliver pivotal long‐term validation and establish a rigorously characterized, clinically translatable platform for personalized cell therapy in hereditary Parkinson's disease.

## 5 Methods

### 5.1 Generation and Gene Correction of Human iPSC Lines

Peripheral blood was obtained from a Parkinson's disease (PD) patient carrying compound heterozygous mutations (R50H and M2397T) in the LRRK2 gene. This procedure was approved by the Institutional Review Board of Sichuan Provincial People's Hospital, Sichuan Academy of Medical Sciences (approval no^#^.: ER(R)‐2021‐0385).

PBMCs were isolated from peripheral blood using Ficoll (G&E Healthcare) and then culture for one week in SFM medium (50%IMDM, 50% Ham's F12, 1% ITS‐X, 1% chemically defined lipid concentrate, 1% Glutamax, 50µg/mL ascorbic acid, 5 mg/mL BSA, 200Μm 1‐thioglycerol) suppled with SCF (100ng/mL; PeproTech), IL‐3 (10ng/mL; PeproTech), EPO (2U/mL; R&D Systems), IGF‐1 (40ng/mL; PeproTech), Dexamethasone (1µm; Sigma–Aldrich) and transferrin (100µg/mL; R&D systems).

Reprogramming was conducted using CytoTune ‐Ips 2.0 Sendai Reprogramming Kit (Invitrogen). Briefly, 1 × 10^5^ cells were infected with three vectors (Klf4‐Oct3/4‐Sox2, cMyc, and Klf4). Two weeks later, colonies were observed. These colonies were split onto Matrigel‐coated 6‐well plate and maintained in mTeSR (Stem Cell Technologies) medium. After several passages, homogeneous colonies were generated.

Pluripotency was validated by immunostaining (NANOG, OCT4, TRA‐1‐60), flow cytometry (SSEA4, TRA‐1‐60), and in vitro trilineage differentiation via embryoid body (EB) formation, with confirmation of ectoderm, mesoderm, and endoderm markers. Gene correction of both LRRK2 variants was performed using the Alt‐R CRISPR‐Cas9 system (Integrated DNA Technologies) combined with PE3 prime editing (Addgene plasmids #132775, #132776).

### 5.2 Sanger Sequencing of hiPSC Line

Allelic correction, potential off‐target effects, and residual plasmid integration in prime‐edited hiPSC lines were assessed by Sanger sequencing. Briefly, genomic DNA was extracted from both edited and unedited control hiPSCs. Targeted PCR amplification was employed on genomic loci of interest, followed by Sanger sequencing. Sequence traces were analyzed for the presence of heterozygous peaks, unexpected insertions/deletions, or plasmid‐derived sequences.

### 5.3 Karyotype Analysis of hiPSC Line

Karyotype analysis was performed on both the parental mutant hiPSC line and the gene‐corrected hiPSC line to assess genomic stability. Cells were treated with colcemid to arrest them in metaphase, followed by hypotonic treatment and fixation. Chromosomes were then stained using G‐banding, and metaphase spreads were imaged and analyzed. At least 20 metaphase cells were examined per line to identify numerical or structural chromosomal abnormalities.

### 5.4 Differentiation Into Midbrain Dopaminergic Progenitors

Directed differentiation followed a three‐stage protocol. Stage 1: Dual‐SMAD inhibition with Y‐27632 (10µm), SB431542 (SB; 10 mm; Sigma), Noggin (100ng/mL; Miltenyi), Shh‐C24II (300ng/mL; Myltenyi), CHIR99021 (1µm; Tocris), and Purmorphamine (Pur, 0.5µm; Calbiochem) in N2 media (DMEM/F12, Neurobasal, 1XN2, 1XGlutamax) for neural induction from day 0 till day 9. Stage 2: Patterning with FGF8b (100 ng/mL, Peprotech) in B27 medium (Neurobasal, 2% B27 without vitamin A, 1X Glutamax) for 8 days. Stage 3: Maturation with BDNF (20 ng/mL, Peprotech), GDNF (20 ng/mL, Peprotech), L‐ascorbic acid (L‐AA, 200 µm; Sigma), and dibutyryl‐cAMP (0.5 µm; Sigma) in B27 medium up to 40 days. Marker expression was assessed on day 16 (LMX1, FOXA2, NESTIN) and day 50 (TH, MAP2, SYN1, PSD95) via immunocytochemistry and flow cytometry.

### 5.5 Immunohistochemistry of Dopaminergic Progenitors

For immunocytochemical analysis, dopaminergic progenitors were seeded onto glass coverslips and fixed at the indicated time points. Cells were immunostained with anti‐LMX1A antibody (Abcam, ab309538), FOXA2 antibody (Cell Signaling, 8186S), and counterstained with Hoechst 33342 (dilution; Invitrogen, H21492) to visualize nuclei. Immunofluorescence images were acquired using a Nikon Eclipse TE2000‐S fluorescence microscope (Nikon Instruments, Sterling Heights, MI, USA). For quantitative analysis, the percentages of LMX1A^+^, FOXA2^+^, and LMX1A^+^/FOXA2^+^ double‐positive cells relative to total Hoechst^+^ cells were counted using ImageJ software (National Institutes of Health, Bethesda, MD, USA). Data are presented as mean ± standard deviation (SD).

### 5.6 Assay of Dopamine by ELISA

Dopamine release was quantified by enzyme‐linked immunosorbent assay (ELISA, Sangon Biotech, D751019). Briefly, differentiated dopaminergic neurons were seeded onto Matrigel‐coated culture plates, and dopamine release was assessed between days 35 and 50 post‐differentiation. Prior to stimulation, cells were gently washed with physiological saline solutions. High‐potassium depolarization was induced by adding 60 mM KCl, and cells were incubated at 37°C with 5% CO_2_ for 30 min. The conditioned medium was subsequently collected, and dopamine concentrations were determined using a commercially available ELISA kit following the manufacturer's protocol.

### 5.7 Non‐Human Primate PD Model

All procedures involving animals were performed at the Institute of Zoology, Guangdong Academy of Sciences, a nonhuman primate research facility in line with AAALAC‐accredited. All animal protocols were approved by the animal care and the Committee of the Institute of Zoology, Guangdong Academy of Sciences (approval no.: GIZ20210510). Twelve aged male cynomolgus monkeys (～13 years old) were housed in social pairs with enrichment, in line with ARRIVE guidelines, and monitored by veterinary staff.

To create Parkinsonian models, nine monkeys were subcutaneously administered MPTP continuously via subcutaneous ALZET osmotic pumps (model 2ML4; 0.1 mg/kg/day for 28 days). Post‐lesion stabilization was monitored via behavioral scoring and body weight tracking. The ALZET osmotic pump was removed when persistent Parkinsonian symptoms were observed, such as tremors, rigidity, bradykinesia, and impaired balance.

The PD monkey models were randomly divided into three groups. (group1: ^#^090025, ^#^072289, ^#^072197, DA‐NPC1 grafts; group2: ^#^081459, ^#^061307, ^#^091713, DA‐NPC2 grafts; group3: ^#^091489, ^#^085175, ^#^056507 for CSF control). Three monkeys were selected as age‐matched healthy controls (group 4: ^#^07649, ^#^086259, ^#^071475).

### 5.8 Cell Transplantation

Since the caudate nucleus and putamen nucleus in the striatum are the main entry points for receiving dopamine signals [71], cell transplantation is carried out in these regions of the striatum using an MRI‐guided stereotactic frame (Crist Instruments). Before the transplantation surgery was carried out, the PD cynomolgus monkeys for DN cells transplantation underwent a cranial MR scan. Based on the MRI scan data and in combination with the Atlas of the Rhesus Monkey Brain, the injection target coordinates for the animals were determined by the ear‐canthal plane and the midline as reference points.

Prior to in vivo transplantation, we performed an in vitro preliminary viability assay to evaluate the shear stress induced during the cell aspiration–dispensing cycle of the Hamilton syringe. Briefly, cells were aspirated into and ejected out of the injection syringe sequentially, and cell viability was quantified via trypan blue exclusion before and after the whole procedure.

The transplanted dopaminergic progenitor cells exhibited an average diameter of 11µm and were formulated to a final concentration of 50 000 cells/µL for transplantation. After craniotomy, a 28‐G Hamilton syringe (inner diameter: 184 µm) was fixed on the stereotactic apparatus. Under the precise guidance of the stereotactic apparatus, a DN cell suspension at a concentration of 50 000 cells/µL was delivered at a rate of 1µL/min over 10 min using a gauge‐28 Hamilton needle controlled by a peristaltic pump. In detail, 10 µL was delivered stereotactically at 1 µL/min into each of the caudate nucleus and putamen per hemisphere over 10 min. A 10‐min dwell period followed by infusion to promote local pressure equilibration and prevent retrograde backflow along the needle tract.

After surgery, monkeys were given antibiotics for 3 days. Immunosuppression (Cyclosporine A, 10 mg/kg/day) was initiated two days prior to transplantation and maintained until the end of the study endpoint. PD model monkeys receiving identical surgical procedures and 10 µL acellular CSF per injection site served as vehicle controls.

### 5.9 PET‐CT Imaging

PET/CT imaging was performed using the uEXPLORER total‐body PET/CT Scanner (Shanghai, China) at 18 months post‐grafting in all animals, with control animals scanned at matched time points to ensure consistent acquisition and comparability. To monitor dopaminergic function in monkey brains transplanted with iPSC‐derived DA‐NPC cells, in vivo PET imaging was conducted prior to euthanasia. Monkeys were initially sedated by intramuscular injection of Zoletil 50 (0.1 mL/kg), followed by intravenous administration of pentobarbital (0.2–0.5 mL/kg) to achieve stable and deep anesthesia. PET tracer [^18^F] DOPA (at a dose of 37 MBq/kg) was synthesized under GMP conditions and administered intravenously. Dynamic PET scans were acquired for 120 min using a Siemens Biograph PET/CT scanner. Data were analyzed using PMOD software (ROI definition and time‐activity curves). Semi‐quantitative analysis of 18F‐DOPA uptake was performed using standardized uptake values (SUVs). The striatum, including the caudate nucleus and putamen, was manually delineated as the region of interest (ROI) on coregistered PET/CT images. SUV was calculated as tissue radioactivity concentration (kBq/mL) divided by injected activity (MBq) normalized to body weight (kg). To mitigate inter‐animal variability, the cerebellar cortex was used as the reference region, and a normalized uptake index (UI) was calculated as: UI = (SUV_ROI / SUV_Cerebellum) − 1. The one‐way ANOVA was performed, followed by Tukey's multiple comparisons test to compare differences among groups.

### 5.10 MRI Scans

MRI scans were acquired at Pre‐MPTP, Pre‐grafting, and quarterly intervals post‐grafting up to 18 months using a 3T GE Discovery MR750 scanner (Milwaukee, WI, USA) with T1‐ and T2‐weighted fast spin‐echo sequences. These acquisitions enabled longitudinal monitoring of graft localization, striatal tissue volume, and potential adverse structural changes.

### 5.11 Tissue Preparation

18 months after cell transplantation, 10 of the 12 monkeys were deeply anesthetized with an overdose of isoflurane and pentobarbital and perfused with 2 liters of cold carbogen‐bubbled artificial cerebrospinal fluid (ASCF) to reduce the internal brain temperature gradually.

The entire corpus striatum was removed and immersed in fresh ice‐cold carbogen‐bubbled N‐methyl‐D glucamine (NMDG) solution. Unilateral striata from DA‐NPCs groups and controls were sectioned into 300 µm thick slices using a vibratome (Leica VT 1200S) in ice‐cold NMDG solution bubbled with 95% O_2_/5% CO_2_ at 34°C for 10 mins.

The other side of the striatum was immersed in 4% ice‐cold phosphate‐buffered paraformaldehyde (PFA) for 24 h. The tissue was then resuspended in a graded 30% sucrose solution and sliced into 40‐µm serial sections on a freezing microtome (Leica SM2020R) and stored at −20 °C in a cryoprotectant solution.

### 5.12 Brain Slice and Electrophysiological Recording

The acute brain slices for electrophysiological recordings were prepared following the previously described protocol [72]. Briefly, the tissue block containing the putamen was rapidly removed from the animal brain and immersed in ice‐cold (0°C–4°C) oxygenated NMDG (N‐methyl‐d‐glucamine) solution containing (in mm) 93 mm NMDG, 93 mm HCl, 2.5 mm KCl, 1.2 mm NaH_2_PO_4_, 30 mm NaHCO_3_, 20 mm HEPES, 25 mm glucose, 5 mm sodium ascorbate, 2 mm thiourea, 3 mm sodium pyruvate, 10 mM MgSO_4_, 0.5 mm CaCl_2_, and finally adjusted the pH value to 7.35 with HCl (Sigma–Aldrich). 300‐µm‐thick coronal slices were obtained using a Leica microtome (Leica, VT1200s, Leica Biosystems, Germany). After incubation in oxygenated NMDG solution at 34.0 ± 0.5°C for 10–15 min, the slices were transferred to artificial cerebrospinal fluid (ACSF) solution (125 mm NaCl, 2.5 mm KCl, 1.25 mm NaH_2_PO_4_, 25 mm NaHCO_3_, 1 mm MgCl_2_, 11.1 mM glucose and 2 mm CaCl_2_, pH 7.4, all from Sigma–Aldrich) at 34.0 ± 0.5°C for 50 min. Finally, the slices were kept in a chamber submerged in ACSF at room temperature (∼26°C) and continuously oxygenated until the whole‐cell recording was performed.

Whole‐cell patch‐clamp electrophysiological recordings were conducted at room temperature. The patch recording pipettes (5–7 MΩ) were filled with intracellular solution consisting of 120 mm potassium gluconate, 10 mm HEPES, 4 mm KCl, 4 mm MgATP, 0.3 mm Na_3_GTP, 10 mm sodium phosphocreatine, and 0.5% biocytin, adjusted to pH 7.25 using HCl. Fluorescence‐labeled putamen neurons were targeted and recorded. The neuronal signals were amplified using a Quadro EPC 10 (HEKA Electronics, Lambrecht, Germany) and monitored online with PatchMaster (HEKA). We employed the specific quality control criteria: (1) seal resistance value >1 GΩ before achieving whole‐cell configuration; (2) access resistance <30 MΩ. Each neuron was injected with 600‐ms‐long current pulses ranging from −200 to 1380 pA with 20‐pA increment steps, with an interval of 1.5 s. Typically, the recordings were repeated 3–5 times, and the electrophysiological traces obtained approximately 5 min after achieving whole‐cell configuration were selected for analysis. For each neuron, the electrophysiological recording lasted 10–30 min, including EPSC recording sessions, to enable biocytin diffusion into the distal neurite segments.

In some cases, TTX (1 µm) was applied to the external solution to block the sodium channels in the neuronal membrane following the above recordings. After TTX had diffused in the external solution (∼10 min), the electrophysiological recording was performed again, and the recording data were compared with those previously obtained from the same neurons.

The spontaneous excitatory postsynaptic currents (sEPSCs) were collected during the patch recording experiments. The pipette solution contains (in mM): 120 Cs gluconate, 10 HEPES, 4 KCl, 4 MgATP, 0.3 Na_3_GTP, 10 sodium phosphocreatine, and 0.5% biocytin (PH 7.25). After the potential signal had stabilized (showing no significant signal shifts beyond ±4SD of the baseline), EPSCs were recorded while holding the membrane potential at −70 mV. Each trace was recorded for a minimum of 5 min, and the sample traces were plotted using the custom‐made MATLAB routines.

Immediately following the recording, the recorded brain slices were fixed overnight with 4% PFA at 4 °C. To verify the biocytin‐labeled neurons, the slices filled with biocytin (during electrophysiological recordings) were incubated with streptavidin‐Alexa 647 (Invitrogen, S32357), rabbit anti‐TH (Millipore, AB152) and chicken anti‐GFP (abcam, ab13970) at 4 for 72 h. Sections were incubated with corresponding fluorescent secondary antibodies and Hoechst for 2 h at room temperature. The sections were mounted by Fluoromount‐G. Brain slices were imaged using a Leica TCS‐SP5 laser‐scanning confocal microscope (Leica, Germany).

We developed a custom‐made software kit (PatchElectroLab) for offline analysis of electrophysiology data using MATLAB (MATLAB, Mathworks, MA). Neuronal electrophysiological properties were automatically extracted employing a methodology similar to previous studies [66, 72].

### 5.13 Immunohistochemistry of Grafted Cell Phenotype

Immunofluorescence staining was employed to detect and quantify the survival and phenotypic identity of grafted cells. Primary antibodies used in the present study included Chicken anti‐GFP (abcam, ab13970), rabbit anti‐TH (Millipore, AB152), and Goat anti‐GIRK2 (abcam, ab65096). Free‐floating sections were incubated in a blocking buffer (10% donkey serum and 0.2% Triton X‐100 in phosphate buffer saline) for 60 min at room temperature before being incubated in the primary antibodies overnight at 4°C. Fluorescently conjugated secondary antibodies were used to reveal the binding of primary antibodies (1:2000, Invitrogen), and nuclei were stained with Hoechst (Invitrogen H21492). Negative controls without primary antibody were performed in all experiments to monitor nonspecific staining. The immunofluorescent samples were visualized using a Nikon‐Eclipse TE 2000‐S fluorescence microscope (Nikon Instruments, Sterling Heights, MI, USA). To quantify the population of TH and GIRK2 expressing cells among total GFP or GIRK2 to total TH cells, single or double‐stained cells were counted manually with Image J. Data were presented as a ratio of TH‐, GIRK2‐ to total TH/GFP or GIRK2/TH cells. All data are expressed as mean ± SD.

### 5.14 Immunohistochemistry of Host Astrocytic Response

Astrocytic response was assessed by immunofluorescence staining for glial fibrillary acidic protein (GFAP). Briefly, cryosections of striatal tissue from control, L‐Mut‐DA‐NPC, and L‐GC‐DA‐NPC groups were permeabilized and blocked, then incubated with primary antibodies against GFAP (astrocyte marker) and GFP (to identify graft‐derived cells). Sections were then incubated with fluorophore‐conjugated secondary antibodies, and nuclei were counterstained with Hoechst 33342. Images were acquired using a fluorescence microscope under identical exposure parameters. The relative GFAP immunoreactive intensity in the striatal region was quantified using ImageJ software, normalized to the control group, and presented as relative intensity values.

### 5.15 Flow Cytometry

Cells were detached with Accutase solution, and the fixation/permeabilization procedure was followed by BD Cytofix/Cytoperm kit (BD Pharmingen). The percentage of pluripotent stem cells was calculated by staining the OCT4, NANOG, TRA‐1‐60, and SSEA‐4 antibodies; the DA NPCs makers were calculated by staining the LMX1 and FOXA2. Finally, the data were analyzed with flowjo software, and trans‐differentiation efficiency was calculated.

### 5.16 Behavioral Analysis

In brief, each monkey was placed in a transparent cage (1.17 ×1.2 ×0.82 m) alone and a quiet room for video recording (90 min) prior to modelling and the first month after MPTP administration, as well as every month after cell transplantation until the end time point. Video recordings of all monkeys were analyzed using a commercial primate behavior analysis system (PrimateScan 1.0, CleverSys Inc, USA). Movement parameters, including distance, velocity, quadrant preference, and vertical activity, were extracted at multiple time points. Top‐quadrant activity was interpreted as indicative of non‐motor exploratory behavior. Data were normalized to pre‐MPTP baseline levels. The *H&Y* scores were conducted once weekly until persistent and overt Parkinsonian symptoms were observed, and then once a month throughout the testing period. The *H&Y* scores were evaluated blindly by two well‐trained technicians in the following categories (Table S8). All monkeys were initially assessed as neurologically healthy, with all *H&Y* scores equal to 0 prior to MPTP administration. They were confirmed as PD monkeys based on the total *H&Y* score being no less than 8, subsequently.

### 5.17 Systemic Safety Assays

Blood samples were collected at baseline, pre‐transplant, and at sacrifice. Serum was analyzed for tumor markers α‐fetoprotein (AFP), carcinoembryonic antigen (CEA), and neuron‐specific enolase (NSE) using Roche Cobas e411 immunoassays. Liver enzymes alanine aminotransferase (ALT) and aspartate aminotransferase (AST), renal function blood urea nitrogen (BUN) and creatinine (CRE), and lactate dehydrogenase (LDH) were measured on the Beckman Coulter AU5800 analyzer (Brea, CA). Hematology was performed using Sysmex XN‐100 (Kobe, Japan).

### 5.18 Statistical Analysis

All experiments were conducted with blinded analysis and randomized assignments where feasible. Data are expressed as mean ± SD or SEM. Comparisons were made using one‐way or two‐way ANOVA with post hoc Tukey or Bonferroni tests. Repeated measures ANOVA was used for longitudinal PET and behavior data. Statistical analyses were performed in GraphPad Prism v9.5 (San Diego, CA). P < 0.05 was considered statistically significant.

## Author Contributions

Conceptualization, Q.Y, C.X, Q.W, J.G, S.L, R.L, L.Z, and J.R; methodology, Q.Y, C.X, Q.W, J. G, P.W, Q.W, B.L, Z.S, M.S, Y.Z.; software, B.L, J.C, X.S., S.W, X.Z, Y.J.; wet laboratory experiments: Q.Y, C.X, Q.W, J.G, R.L, P.W, M.L, B.L, H.A, N.X, Y.L, and L.T.; assisted by M.H, N.Y, Y.S, F.W, X.Z. M.Z, J.C, Z.W, and X. L; data analysis, C.X, Q.W, J.G, Y.J, and X.Z; writing – original draft preparation, Q.Y, C.X, Q.W, P.W, J.G, and Y.J; writing – review and editing, S.L, R.L, L.Z, and J.R. All authors have read and agreed to the published version of the manuscript.

## Ethics Statement

The collection of human peripheral blood samples was approved by the Institutional Review Board of Sichuan Provincial People's Hospital, Sichuan Academy of Medical Sciences (approval no.^#^: ER(R)‐2021‐0385). All animal experimental protocols were approved by the Animal Care and Use Committee of the Institute of Zoology, Guangdong Academy of Sciences (approval no.^#^: GIZ20210510), and were conducted in accordance with the guidelines for the care and use of laboratory animals.

## Consent

Following written informed consent, peripheral blood was obtained from a Parkinson's disease patient harboring compound heterozygous LRRK2 mutations (R50H and M2397T) and a healthy volunteer for research use.

## Conflicts of Interest

The authors declare no conflicts of interest.

## Supporting information




**Supporting File**: advs76394‐sup‐0001‐SuppMat.docx.

## Data Availability

All data generated or analyzed during this study are included in this published article and its supplementary files or are available upon request. No custom software, proprietary algorithms, or external databases were used in data acquisition, processing, or analysis.

## References

[1] D. J. Moore , A. B. West , V. L. Dawson , and T. M. Dawson , “Molecular Pathophysiology of Parkinson's Disease,” Annual Review of Neuroscience 28 (2005): 57–87. 10.1146/annurev.neuro.28.061604.135718 16022590

[2] B. R. Bloem , M. S. Okun , and C. Klein , “Parkinson's Disease,” The Lancet 397, no. 10291 (2021): 2284–2303. 10.1016/S0140-6736(21)00218-X 33848468

[3] J. Jankovic , “Parkinson's Disease: Clinical Features and Diagnosis,” Journal of Neurology, Neurosurgery & Psychiatry 79, no. 4 (2008): 368–376. 10.1136/jnnp.2007.131045 18344392

[4] L. V. Kalia and A. E. Lang , “Parkinson's Disease,” The Lancet 386, no. 9996 (2015): 896–912. 10.1016/S0140-6736(14)61393-3 25904081

[5] L. Studer , “Derivation of Dopaminergic Neurons From Pluripotent Stem Cells,” Progress in Brain Research 200 (2012): 243–263. 10.1016/B978-0-444-59575-1.00011-9 23195422

[6] V. Tabar and L. Studer , “Pluripotent Stem Cells in Regenerative Medicine: Challenges and Recent Progress,” Nature Reviews Genetics 15, no. 2 (2014): 82–92. 10.1038/nrg3563 PMC4539940 24434846

[7] R. A. Barker , J. Drouin‐Ouellet , and M. Parmar , “Cell‐Based Therapies for Parkinson Disease—Past Insights and Future Potential,” Nature Reviews Neurology 11, no. 9 (2015): 492–503. 10.1038/nrneurol.2015.123 26240036

[8] O. Lindvall and Z. Kokaia , “Stem Cells for the Treatment of Neurological Disorders,” Nature 441, no. 7097 (2006): 1094–1096. 10.1038/nature04960 16810245

[9] A. Björklund and O. Lindvall , “Cell Replacement Therapies for Central Nervous System Disorders,” Nature Neuroscience 3, no. 6 (2000): 537–544. 10.1038/75705 10816308

[10] K. Takahashi and S. Yamanaka , “Induction of Pluripotent Stem Cells From Mouse Embryonic and Adult Fibroblast Cultures by Defined Factors,” Cell 126, no. 4 (2006): 663–676. 10.1016/j.cell.2006.07.024 16904174

[11] K. Takahashi , K. Tanabe , M. Ohnuki , et al., “Induction of Pluripotent Stem Cells From Adult Human Fibroblasts by Defined Factors,” Cell 131, no. 5 (2007): 861–872. 10.1016/j.cell.2007.11.019 18035408

[12] J. Yu , M. A. Vodyanik , K. Smuga‐Otto , et al., “Induced Pluripotent Stem Cell Lines Derived From Human Somatic Cells,” Science 318, no. 5858 (2007): 1917–1920. 10.1126/science.1151526 18029452

[13] N. Fusaki , H. Ban , A. Nishiyama , K. Saeki , and M. Hasegawa , “Efficient Induction of Transgene‐Free Human Pluripotent Stem Cells Using a Vector Based on Sendai Virus, an RNA Virus That Does not Integrate Into the Host Genome,” Proceedings of the Japan Academy, Series B 85, no. 8 (2009): 348–362. 10.2183/pjab.85.348 PMC3621571 19838014

[14] H. Ban , N. Nishishita , N. Fusaki , et al., “Efficient Generation of Transgene‐Free Human Induced Pluripotent Stem Cells (iPSCs) by Temperature‐Sensitive Sendai Virus Vectors,” Proceedings of the National Academy of Sciences 108, no. 34 (2011): 14234–14239. 10.1073/pnas.1103509108 PMC3161531 21821793

[15] S. M. Chambers , C. A. Fasano , E. P. Papapetrou , M. Tomishima , M. Sadelain , and L. Studer , “Highly Efficient Neural Conversion of Human ES and iPS Cells by Dual Inhibition of SMAD Signaling,” Nature Biotechnology 27, no. 3 (2009): 275–280. 10.1038/nbt.1529 PMC2756723 19252484

[16] C. R. Freed , P. E. Greene , R. E. Breeze , et al., “Transplantation of Embryonic Dopamine Neurons for Severe Parkinson's Disease,” New England Journal of Medicine 344, no. 10 (2001): 710–719. 10.1056/NEJM200103083441002 11236774

[17] J. S. Schweitzer , B. Song , T. M. Herrington , et al., “Personalized iPSC‐Derived Dopamine Progenitor Cells for Parkinson's Disease,” New England Journal of Medicine 382, no. 20 (2020): 1926–1932. 10.1056/NEJMoa1915872 PMC7288982 32402162

[18] T. W. Kim , S. Y. Koo , and L. Studer , “Pluripotent Stem Cell Therapies for Parkinson Disease: Present Challenges and Future Opportunities,” Frontiers in Cell and Developmental Biology 8 (2020): 729. 10.3389/fcell.2020.00729 PMC7438741 32903681

[19] J. Takahashi , “iPS Cell‐Based Therapy for Parkinson's Disease: A Kyoto Trial,” Regenerative Therapy 13 (2020): 18–22. 10.1016/j.reth.2020.06.002 PMC7794047 33490319

[20] N. Sawamoto , D. Doi , E. Nakanishi , et al., “Phase I/II Trial of iPS‐Cell‐Derived Dopaminergic Cells for Parkinson's Disease,” Nature 641, no. 8064 (2025): 971–977. 10.1038/s41586-025-08700-0 PMC12095070 40240591

[21] J. W. Chang , H. K. Na , K. W. Chang , et al., “Phase 1/2a Clinical Trial of hESC‐Derived Dopamine Progenitors in Parkinson's Disease,” Cell 188, no. 25 (2025): 7036–7048.e11. 10.1016/j.cell.2025.09.010 41086804

[22] A. Zimprich , S. Biskup , P. Leitner , et al., “Mutations in LRRK2 Cause Autosomal‐Dominant Parkinsonism with Pleomorphic Pathology,” Neuron 44, no. 4 (2004): 601–607. 10.1016/j.neuron.2004.11.005 15541309

[23] C. Paisán‐Ruíz , S. Jain , E. W. Evans , et al., “Cloning of the Gene Containing Mutations That Cause PARK8‐Linked Parkinson's Disease,” Neuron 44, no. 4 (2004): 595–600. 10.1016/j.neuron.2004.10.023 15541308

[24] D. G. Healy , M. Falchi , S. S. O'Sullivan , et al., “Phenotype, Genotype, and Worldwide Genetic Penetrance of LRRK2‐Associated Parkinson's Disease: A Case‐Control Study,” The Lancet Neurology 7, no. 7 (2008): 583–590. 10.1016/S1474-4422(08)70117-0 PMC2832754 18539534

[25] E. Greggio and M. R. Cookson , “Leucine‐Rich Repeat Kinase 2 Mutations and Parkinson's Disease: Three Questions,” ASN Neuro 1, no. 1 (2009): AN20090007. 10.1042/AN20090007 PMC2695577 19570025

[26] J. C. Dächsel and M. J. Farrer , “LRRK2 and Parkinson Disease,” Archives of Neurology 67, no. 5 (2010): 542–547. 10.1001/archneurol.2010.79 20457952

[27] M. R. Cookson , “LRRK2 Pathways Leading to Neurodegeneration,” Current Neurology and Neuroscience Reports 15, no. 7 (2015): 42. 10.1007/s11910-015-0564-y PMC5839465 26008812

[28] I. F. Mata , W. J. Wedemeyer , M. J. Farrer , J. P. Taylor , and K. A. Gallo , “LRRK2 in Parkinson's Disease: Protein Domains and Functional Insights,” Trends in Neurosciences 29, no. 5 (2006): 286–293. 10.1016/j.tins.2006.03.006 16616379

[29] M. Jinek , K. Chylinski , I. Fonfara , M. Hauer , J. A. Doudna , and E. Charpentier , “A Programmable Dual‐RNA–Guided DNA Endonuclease in Adaptive Bacterial Immunity,” Science 337, no. 6096 (2012): 816–821. 10.1126/science.1225829 PMC6286148 22745249

[30] L. Cong , F. A. Ran , D. Cox , et al., “Multiplex Genome Engineering Using CRISPR/Cas Systems,” Science 339, no. 6121 (2013): 819–823. 10.1126/science.1231143 PMC3795411 23287718

[31] P. Mali , L. Yang , K. M. Esvelt , et al., “RNA‐Guided Human Genome Engineering via Cas9,” Science 339, no. 6121 (2013): 823–826. 10.1126/science.1232033 PMC3712628 23287722

[32] A. C. Komor , Y. B. Kim , M. S. Packer , J. A. Zuris , and D. R. Liu , “Programmable Editing of a Target Base in Genomic DNA Without Double‐Stranded DNA Cleavage,” Nature 533, no. 7603 (2016): 420–424. 10.1038/nature17946 PMC4873371 27096365

[33] N. M. Gaudelli , A. C. Komor , H. A. Rees , et al., “Programmable Base Editing of A•T to G•C in Genomic DNA Without DNA Cleavage,” Nature 551, no. 7681 (2017): 464–471. 10.1038/nature24644 PMC5726555 29160308

[34] A. V. Anzalone , P. B. Randolph , J. R. Davis , et al., “Search‐and‐Replace Genome Editing Without Double‐Strand Breaks or Donor DNA,” Nature 576, no. 7785 (2019): 149–157. 10.1038/s41586-019-1711-4 PMC6907074 31634902

[35] P. D. Hsu , E. S. Lander , and F. Zhang , “Development and Applications of CRISPR‐Cas9 for Genome Engineering,” Cell 157, no. 6 (2014): 1262–1278. 10.1016/j.cell.2014.05.010 PMC4343198 24906146

[36] Y. Xiong and J. Yu , “LRRK2 in Parkinson's Disease: Upstream Regulation and Therapeutic Targeting,” Trends in Molecular Medicine 30, no. 10 (2024): 982–996. 10.1016/j.molmed.2024.07.003 PMC11466701 39153957

[37] L. H. Sanders , J. Laganière , O. Cooper , et al., “LRRK2 Mutations Cause Mitochondrial DNA Damage in iPSC‐Derived Neural Cells From Parkinson's Disease Patients: Reversal by Gene Correction,” Neurobiology of Disease 62 (2014): 381–386. 10.1016/j.nbd.2013.10.013 PMC3877733 24148854

[38] H. N. Nguyen , B. Byers , B. Cord , et al., “LRRK2 Mutant iPSC‐Derived DA Neurons Demonstrate Increased Susceptibility to Oxidative Stress,” Cell Stem Cell 8, no. 3 (2011): 267–280. 10.1016/j.stem.2011.01.013 PMC3578553 21362567

[39] Y. L. Sosero and Z. Gan‐Or , “LRRK2 and Parkinson's Disease: From Genetics to Targeted Therapy,” Annals of Clinical and Translational Neurology 10, no. 6 (2023): 850–864. 10.1002/acn3.51776 PMC10270275 37021623

[40] S. Kriks , J.‐W. Shim , J. Piao , et al., “Dopamine Neurons Derived From Human ES Cells Efficiently Engraft in Animal Models of Parkinson's Disease,” Nature 480, no. 7378 (2011): 547–551. 10.1038/nature10648 PMC3245796 22056989

[41] D. Doi , B. Samata , M. Katsukawa , et al., “Isolation of Human Induced Pluripotent Stem Cell‐Derived Dopaminergic Progenitors by Cell Sorting for Successful Transplantation,” Stem Cell Reports 2, no. 3 (2014): 337–350. 10.1016/j.stemcr.2014.01.013 PMC3964289 24672756

[42] S. Grealish , A. Heuer , T. Cardoso , et al., “Monosynaptic Tracing Using Modified Rabies Virus Reveals Early and Extensive Circuit Integration of Human Embryonic Stem Cell‐Derived Neurons,” Stem Cell Reports 4, no. 6 (2015): 975–983. 10.1016/j.stemcr.2015.04.011 PMC4471831 26004633

[43] D. Doi , H. Magotani , T. Kikuchi , et al., “Pre‐Clinical Study of Induced Pluripotent Stem Cell‐Derived Dopaminergic Progenitor Cells for Parkinson's Disease,” Nature Communications 11, no. 1 (2020): 3369. 10.1038/s41467-020-17165-w PMC7338530 32632153

[44] B. Song , Y. Cha , S. Ko , et al., “Human Autologous iPSC–Derived Dopaminergic Progenitors Restore Motor Function in Parkinson's Disease Models,” Journal of Clinical Investigation 130, no. 2 (2020): 904–920. 10.1172/JCI130767 PMC6994130 31714896

[45] T. Kikuchi , A. Morizane , D. Doi , et al., “Human iPS Cell‐Derived Dopaminergic Neurons Function in a Primate Parkinson's Disease Model,” Nature 548, no. 7669 (2017): 592–596. 10.1038/nature23664 28858313

[46] E. W. Daadi , E. S. Daadi , T. Oh , M. Li , J. Kim , and M. M. Daadi , “Combining Physical & Cognitive Training With iPSC‐Derived Dopaminergic Neuron Transplantation Promotes Graft Integration & Better Functional Outcome in Parkinsonian Marmosets,” Experimental Neurology 374 (2024): 114694. 10.1016/j.expneurol.2024.114694 PMC13102543 38272159

[47] A. López‐Ornelas , et al., “Human Embryonic Stem Cell‐Derived Immature Midbrain Dopaminergic Neurons Transplanted in Parkinsonian Monkeys,” Cells 12, no. 23 (2023): 2738. 10.3390/cells12232738 PMC10706241 38067166

[48] F. Wianny , K. Dzahini , K. Fifel , et al., “Induced Cognitive Impairments Reversed by Grafts of Neural Precursors: A Longitudinal Study in a Macaque Model of Parkinson's Disease,” Advanced Science 9, no. 10 (2022): 2103827. 10.1002/advs.202103827 PMC8981458 35137562

[49] Y. Tao , S. C. Vermilyea , M. Zammit , et al., “Autologous Transplant Therapy Alleviates Motor and Depressive Behaviors in Parkinsonian Monkeys,” Nature Medicine 27, no. 4 (2021): 632–639. 10.1038/s41591-021-01257-1 PMC8198752 33649496

[50] J. W. Langston , P. Ballard , J. W. Tetrud , and I. Irwin , “Chronic Parkinsonism in Humans Due to a Product of Meperidine‐Analog Synthesis,” Science 219, no. 4587 (1983): 979–980. 10.1126/science.6823561 6823561

[51] S. P. Markey , J. N. Johannessen , C. C. Chiueh , R. S. Burns , and M. A. Herkenham , “Intraneuronal Generation of a Pyridinium Metabolite May Cause Drug‐Induced Parkinsonism,” Nature 311, no. 5985 (1984): 464–467. 10.1038/311464a0 6332988

[52] J. Nonnekes , B. Post , J. W. Tetrud , J. W. Langston , and B. R. Bloem , “MPTP‐Induced Parkinsonism: An Historical Case Series,” The Lancet Neurology 17, no. 4 (2018): 300–301. 10.1016/S1474-4422(18)30072-3 29553378

[53] P. Jokinen , H. Helenius , E. Rauhala , A. Brück , O. Eskola , and J. O. Rinne , “Simple Ratio Analysis of 18F‐Fluorodopa Uptake in Striatal Subregions Separates Patients With Early Parkinson Disease From Healthy Controls,” Journal of Nuclear Medicine 50, no. 6 (2009): 893–899. 10.2967/jnumed.108.061572 19443601

[54] D. J. Brooks , V. Ibanez , G. V. Sawle , et al., “Differing Patterns of Striatal 18 F‐Dopa Uptake in Parkinson's Disease, Multiple System Atrophy, and Progressive Supranuclear Palsy,” Annals of Neurology 28, no. 4 (1990): 547–555. 10.1002/ana.410280412 2132742

[55] P. K. Morrish , G. V. Sawle , and D. J. Brooks , “An [ 18 F]dopa–PET and Clinical Study of the Rate of Progression in Parkinson's Disease,” Brain 119, no. 2 (1996): 585–591. 10.1093/brain/119.2.585 8800950

[56] N. Pavese , M. Rivero‐Bosch , S. J. Lewis , A. L. Whone , and D. J. Brooks , “Progression of Monoaminergic Dysfunction in Parkinson's Disease: A Longitudinal 18F‐Dopa PET Study,” Neuroimage 56, no. 3 (2011): 1463–1468. 10.1016/j.neuroimage.2011.03.012 21396455

[57] A. Egerton , A. Demjaha , P. McGuire , M. A. Mehta , and O. D. Howes , “The Test–Retest Reliability of 18F‐DOPA PET in Assessing Striatal and Extrastriatal Presynaptic Dopaminergic Function,” Neuroimage 50, no. 2 (2010): 524–531. 10.1016/j.neuroimage.2009.12.058 PMC4096947 20034580

[58] H. Li , H. Jiang , H. Li , L. Li , Z. Yan , and J. Feng , “Generation of Human A9 Dopaminergic Pacemakers From Induced Pluripotent Stem Cells,” Molecular Psychiatry 27, no. 11 (2022): 4407–4418. 10.1038/s41380-022-01628-1 PMC9684358 35610351

[59] A. K. Mishra , S. Dixit , A. Singh , T. Shukla , and S. I. Rizvi , “Molecular Determinants of A9 Dopaminergic Neurons,” NeuroMolecular Medicine 27, no. 1 (2025): 43. 10.1007/s12017-025-08861-1 40397062

[60] T. Kamath , A. Abdulraouf , S. J. Burris , et al., “Single‐Cell Genomic Profiling of Human Dopamine Neurons Identifies a Population That Selectively Degenerates in Parkinson's Disease,” Nature Neuroscience 25, no. 5 (2022): 588–595. 10.1038/s41593-022-01061-1 PMC9076534 35513515

[61] Z. Yang and K. K. Wang , “Glial Fibrillary Acidic Protein: From Intermediate Filament Assembly and Gliosis to Neurobiomarker,” Trends in Neurosciences 38, no. 6 (2015): 364–374. 10.1016/j.tins.2015.04.003 PMC4559283 25975510

[62] J. P. Heller and D. A. Rusakov , “Morphological Plasticity of Astroglia: Understanding Synaptic Microenvironment,” Glia 63, no. 12 (2015): 2133–2151. 10.1002/glia.22821 PMC4737250 25782611

[63] J. W. Goh , J. L. Lim , T. S. Toh , et al., “LRRK2 p.G2385R and p.R1628P variants in a multi‐ethnic Asian Parkinson's Cohort: Epidemiology and clinical insights,” npj Parkinson's Disease 11, no. 1 (2025): 320. 10.1038/s41531-025-01166-x PMC12627830 41253790

[64] A. Usmani , F. Shavarebi , and A. Hiniker , “The Cell Biology of LRRK2 in Parkinson's Disease,” Molecular and Cellular Biology 41, no. 5 (2021): e00660‐20. 10.1128/MCB.00660-20 PMC8088268 33526455

[65] Y. Wu , A. Zhong , M. Sidharta , et al., “Robust and Inducible Genome Editing Via an All‐in‐One Prime Editor in Human Pluripotent Stem Cells,” Nature Communications 15, no. 1 (2024): 10824. 10.1038/s41467-024-55104-1 PMC11685797 39737975

[66] F. Scala , D. Kobak , M. Bernabucci , et al., “Phenotypic Variation of Transcriptomic Cell Types in Mouse Motor Cortex,” Nature 598, no. 7879 (2021): 144–150. 10.1038/s41586-020-2907-3 PMC8113357 33184512

[67] S. Zhang , L. Yuan , Z. Wu , et al., “Non‐Human Primate Models of Parkinson's Disease: Decoding Pathogenesis and Advancing Therapies,” Brain‐x 3, no. 2 (2025): 70032.

[68] G. J. Masilamoni and Y. Smith , “Chronic MPTP Administration Regimen in Monkeys: A Model of Dopaminergic and Non‐Dopaminergic Cell Loss in Parkinson's Disease,” Journal of Neural Transmission 125, no. 3 (2018): 337–363. 10.1007/s00702-017-1774-z PMC5826821 28861737

[69] R. Tomer , R. Z. Goldstein , G.‐J. Wang , C. Wong , and N. D. Volkow , “Incentive Motivation is Associated With Striatal Dopamine Asymmetry,” Biological Psychology 77, no. 1 (2008): 98–101. 10.1016/j.biopsycho.2007.08.001 PMC2413324 17868972

[70] C. Ren , F. Wang , L.‐N. Guan , et al., “A Compendious Summary of Parkinson's Disease Patient‐Derived iPSCs in the First Decade,” Annals of Translational Medicine 7, no. 22 (2019): 685. 10.21037/atm.2019.11.16 PMC6944564 31930086

[71] J. Li , N. Li , J. Wei , et al., “Genetically Engineered Mesenchymal Stem Cells With Dopamine Synthesis for Parkinson's Disease in Animal Models,” npj Parkinson's Disease 8, no. 1 (2022): 175. 10.1038/s41531-022-00440-6 PMC9780305 36550118

[72] J. R. Wei , Z. Z. Hao , C. Xu , et al., “Identification of Visual Cortex Cell Types and Species Differences Using Single‐Cell RNA Sequencing,” Nature Communications 13, no. 1 (2022): 6902. 10.1038/s41467-022-34590-1 PMC9653448 36371428

