# Supplementary material for: Gene Correction Enhances Dopaminergic Cell Therapy in a Nonhuman Primate Model of Parkinson's Disease
Source: Adv Sci (Weinh). 2026 Jul 6;13(54):e76394. doi: 10.1002/advs.76394 (PMC13334599; doi:10.1002/advs.76394)
Supplement: Supplementary file 1 [file ADVS-13-e76394-s001.docx]

**Gene Correction Enhances Dopaminergic Cell Therapy in a Nonhuman Primate Model of Parkinson`s Disease**

Qing Yan^1, 4#^, Chongchong Xu^2#^, Jiangmei Gao^1#^, Pu Wang^7#^，Qingling Wu^2#^, Ying Jin^1#^, Binyan Lu**^2^**，Mu Li**^2^**, Mingting Shao^3^, Bihai Li^1^，Zhenghui Su^2^，Yijng Zhang^2^，Jianhuan Chen^1, 5^, Haojie An^1^, Mengyao Huang**^3^**，Xiaoji Zhuang**^1^**， Yuhui Shen^3^，Fenglin Wang^6^，Nana Xu^3^，Yiyan Liu^1^, Lei Tang^3^，Xiuling Zhong^1^ , Minyan Zhong^2^, Jiecong Chen^2^, Zhenhuang Wang^2^, Xingrong Luo^2^, Sheng Liu^3*^, Rui-Feng Liu^3*^, Ling Zhang^2 *^, Junhua Rao^1*^

**Figure S1. Characterization of LRRK2-mutant and isogenic control iPSC lines.**

A) Representative immunofluorescence images showing robust expression of pluripotency markers SSEA4 (green) and NANOG (red) in LRRK2-mutant and corrected iPSC colonies. Nuclei are counterstained with Hoechst (blue). Scale bar, 50 μm. B) Flow cytometry analysis confirming high expression of pluripotency surface markers SSEA4 and TRA-1-60 across iPSC lines. C) Immunofluorescence images demonstrating trilineage differentiation potential of iPSCs into cell types of representatives of the three germ layers: βIII-Tubulin (red) for ectoderm/neural lineage, α-SMA (green) for mesoderm, and AFP (magenta) for endoderm. Nuclei counterstained with Hoechst. Scale bar, 50 μm. D) Flow cytometry plots showing co-expression of midbrain progenitor markers LMX1 and FOXA2 following directed differentiation.

**Table S1. Sequences of pegRNAs and nicking gRNAs used for prime editing of PM gene.**

| **Target Mutation** | **Type** | **Sequence (5′→3′)** |
| --- | --- | --- |
| PM M2397T | pegRNA (spacer) | TATACTTTATGGTTCTAGGG |
|  | pegRNA (extension) | GTTTTCTTTTACCATTACCTCCCTAGAACCATAAAG |
|  | Nick gRNA | AGTGTTCTTCTGAAGGCAGA |
| PM R50H | pegRNA (spacer) | GTGTTCACGTACTCCGAGCA |
|  | pegRNA (extension) | AAGTGATTACCGCGCTCGGAGTACGTGA |
|  | Nick gRNA | CAAAATTTGCAAATGTAAGG |

Nucleotide sequences of pegRNAs and nicking gRNAs designed for prime editing-mediated correction of the PM gene mutations M2397T and R50H. Each pegRNA consists of a spacer sequence and a 5′ extension sequence, containing the template for the desired edits. The corresponding nicking gRNAs are also listed.

**Figure S2. Quality control and functional validation of the gene-edited cell.**

(A, B) G-banded karyotyping of the parental mutant hiPSC line (A) and the gene-corrected hiPSC line (B). (C–F) Sanger sequencing chromatograms confirming on-target prime editing of the PM gene mutations. Sequencing traces from unedited control cells (C, E) and gene-corrected cells (D, F) show precise base correction at the target loci (red boxes). (G) Dopamine release assay of L-Mut-DA-NPC and L-GC-DA-NPC. Data are presented as mean ± SD.

**Figure S3. Stereotactic cell transplantation and longitudinal weight tracking in MPTP-lesioned non-human primates.**

A) Illustration of the stereotactic frame and injector system used for precise bilateral transplantation of dopaminergic progenitors into the striatum. The apparatus allows high-precision delivery under MRI guidance. B) Intraoperative image showing transplantation of DA-NPCs into the putamen of a cynomolgus monkey under sterile surgical conditions. C) Stereotactic Brain Coordinates in PD Monkeys Prior to Transplantation. D) Summarizing individual animal body weights from each experimental group at three key time points: prior to MPTP lesioning, one month after MPTP administration, and 18 months post-transplantation. Groups include Group 1: Control; Group 2: Parkinsonian (PD) model with Vehicle transplantation; Group 3: LRRK2-mutant DA-NPC (L-Mut-DA-NPC) recipients; Group 4: Isogenic corrected DA-NPC (L-GC-DA-NPC) recipients. Notably, one animal in Group 3 (ID: 072289) died at 2 months post-transplantation and is excluded from long-term analysis.

**Table S2. Cell death and viability of DA-NPC lines before and after passage through the Hamilton syringe.**

| Cell Line | Baseline dead cell rate (%) | Dead cell rate (%) Post-syringe | Increment of dead cell percentage (%) | Cell viability (%) Post-syringe |
| --- | --- | --- | --- | --- |
| L-Mut-DA-NPC（duplicates） | 4.90 | 7.00 | 2.10 | 93.00 |
| L-GC-DA-NPC（duplicates） | 5.40 | 6.50 | 1.10 | 93.50 |

Quantification of dead cell percentage and post-syringe viability for L-Mut-DA-NPC and L-GC-DA-NPC following aspiration and ejection via a 28-gauge Hamilton syringe. Baseline dead cell percentage reflects initial cell death in the absence of syringe treatment; post-syringe dead cell percentage indicates the proportion of nonviable cells after syringe passage. The increase in dead cell percentage was calculated as the difference between post-syringe and baseline values; post-syringe viability (%) represents the proportion of viable cells remaining post-syringe.

**Figure S4. Longitudinal MRI verification of bilateral graft location at sequential time points.**

MRI scans were acquired at Pre-MPTP, Pre-grafting, 3, 6, 9, 12, 15 and 18 months post-grafting. ND denoted not done.

**Figure S5. [18F] DOPA PET-CT imaging at 18month post-grafting.**

This Figure presents the original image of striatal [¹⁸F]-DOPA uptake for individual animals in each group, which were statistically analyzed and visualized in Figure 2D.

**Table S3. Quantitative data of [¹⁸F] DOPA uptake imaging at 18month post-grafting**

| Goups | Left striatum SUVmean | Right striatum SUVmean | Cerebellum SUVmean | Left striatum relative intensity (Striatum/Cerebellar-1) | Right striatum relative intensity (Striatum/Cerebellar-1) |
| --- | --- | --- | --- | --- | --- |
| CON (07649) | 0.74 | 0.74 | 0.21 | 2.52 | 2.52 |
| CON (086259) | 0.88 | 0.89 | 0.20 | 3.40 | 3.45 |
| PD (056507) | 0.26 | 0.27 | 0.18 | 0.44 | 0.50 |
| PD (091489) | 0.21 | 0.19 | 0.20 | 0.05 | -0.05 |
| L-Mut-DA-NPC (090025) | 0.84 | 0.83 | 0.32 | 1.63 | 1.59 |
| L-Mut-DA-NPC (072197) | 0.47 | 0.47 | 0.19 | 1.47 | 1.47 |
| L-DA-GC-NPC2 (061307) | 0.70 | 0.70 | 0.30 | 0.47 | 0.47 |
| L-DA-GC-NPC 3 (091713) | 0.73 | 0.73 | 0.23 | 2.17 | 2.17 |

This table presents the original numerical data of striatal [¹⁸F]-DOPA uptake for individual animals in each group, which were statistically analyzed and visualized in Figure 2D.

**Figure S6. Assessment of astrocytic response at the graft site in nonhuman primates’ striatum**

A) Representative immunofluorescence images showing GFP⁺ transplanted cells (green) and surrounding GFAP⁺ astrocytes (red) in the striatum. Nuclei are counterstained with Hoechst (blue). Images are shown for control, L-Mut-DA-NPC, and L-GC-DA-NPC groups. Scale bar, 50 μm. B) Quantification of GFAP immunoreactivity as a measure of astrocytic activation across the three experimental groups. Relative GFAP intensity did not differ significantly between control, LRRK2-mutant, and isogenic corrected graft recipients. Data is presented as mean ± SD.

**Table S4. Basic electrophysiological characteristics of grafted human dopamine neurons at 18 months post-transplantation Related to Figure 4.**

| Metric | L-Mut-DA (ID:090025) n = 12 | L-Mut-DA (ID:072197)  n = 16 | L-GC-DA (ID:081459)  n = 19 | L-GC-DA (ID:061307)  n = 19 | L-GC-DA (ID:091713)  n = 17 |
| --- | --- | --- | --- | --- | --- |
| Membrane time constant (Tau, ms) | 7.40 ± 1.38 | 9.03 ± 1.34 | 7.37 ± 1.43 | 10.43 ± 2.47 | 5.66 ± 0.87 |
| Rest membrane potential (mV) | -69.70 ± 0.88 | -69.72 ± 2.84 | -70 ± 1.50 | -68.09 ± 1.57 | -71.97 ± 1.56 |
| AP threshold (mV) | -50.30 ± 2.81 | -46.37 ± 2.16 | -52.36 ± 1.34 | -50.51 ± 2.51 | -51.42 ± 1.80 |
| AP amplitude (mV) | 85.51 ± 5.58 | 89.55 ± 2.96 | 83.45 ± 3.71 | 88.00 ± 2.55 | 93.81 ± 2.39 |
| Membrane input resistance (MΩcm) | 141.85 ± 21.12 | 130.24 ± 19.42 | 126.69 ± 14.42 | 137.18 ± 18.41 | 134.15 ± 13.21 |
| AP half width (ms) | 0.96 ± 0.15 | 1.12 ± 0.11 | 1.28 ± 0.47 | 0.91 ± 0.09 | 0.96 ± 0.15 |
| Sag ratio | 1.30 ± 0.16 | 1.16 ± 0.06 | 1.08 ± 0.01 | 1.13 ± 0.05 | 1.10 ± 0.02 |
| Afterhyperpolarization (mV) | -19.42 ± 5.15 | -14.04 ± 1.26 | -12.68 ± 1.09 | -12.89 ± 2.14 | -13.46 ± 1.15 |
| Max firing rate (Hz) | 62.08 ± 16.46 | 49.90 ± 8.84 | 95.09 ± 12.82 | 60.79 ± 12.42 | 80.00 ± 12.66 |

Summary table of intrinsic membrane properties measured from grafted GFP⁺ human dopamine neurons in the striatum of transplanted primates at 18 months post-transplantation across animals (columns). The values were calculated from whole-cell patch-clamp electrophysiology data derived from TH⁺ neurons and are presented as mean ± SE. The parameters were calculated following the method from published studies.^[64]^ Results indicate preserved electrophysiological function in both LRRK2-mutant and gene-corrected DA-NPC grafts. No statistically significant differences were observed between the two groups, suggesting comparable maturation and integration profiles (p = 0.37, two-way ANOVA with F (1,11) = 0.81).

**Table S5. The Hoehn-Yahr Scoring during this study**

| Group No. | L-mut-DA-NPC1 | L-mut-DA-NPC2 | L-mut-DA-NPC3 | L-GC-DA-NPC1 | L-GC-DA-NPC2 | L-GC-DA-NPC3 | PD | PD | PD | Control 1 | Control 2 |
| --- | --- | --- | --- | --- | --- | --- | --- | --- | --- | --- | --- |
| Animal ID | 090025 | 072289 | 072197 | 081459 | 061307 | 091713 | 091489 | 085175 | 056507 | 07649 | 086529 |
| Pre-MPTP | 0 | 0 | 0 | 0 | 0 | 0 | 0 | 0 | 0 | 0 | 0 |
| PD | 10.00 | 16.00 | 23.00 | 10.00 | 11.00 | 19.00 | 18.00 | 10.00 | 9.00 | 0 | 0 |
| Pre-grafting | 11.00 | 18.50 | 19.00 | 18.00 | 14.50 | 18.50 | 21.50 | 12.50 | 11.00 | 0 | 0 |
| Post-grafting-3M | 0 | Dead at 2M post-grafting | 0 | 11.50 | 0 | 0.50 | 21.00 | 12.50 | 11.00 | 0 | 0 |
| Post-grafting-6M | 0 | ND | 0 | 10.00 | 0 | 0 | 17.00 | 12.50 | 16.50 | 0 | 0 |
| Post-grafting-9M | 0 | ND | 0 | 8.50 | 0 | 0 | 13.50 | 10.50 | 13.50 | 0 | 0 |
| Post-grafting-12M | 0 | ND | 0 | 5.00 | 0 | 3.00 | 18.00 | 10.00 | 10.00 | 0 | 0 |
| Post-grafting-15M | 0 | ND | 0 | 5.50 | 0 | 0 | 17.00 | 10.00 | 8.50 | 0 | 0 |
| Post-grafting-18M | 0 | ND | 0 | 7.00 | 0 | 0 | 20.50 | 8.00 | 8.50 | 0 | 0 |

Pre-MPTP: time point before MPTP lesion establishment; PD: stable Parkinsonian state after MPTP modeling; Pre-grafting: time point prior to cell transplantation; Post-grafting-XM: X months after cell grafting; ND: no data since animal died at 2 months post-transplantation.

**Table S6. Raw quantitative data for spontaneous exploratory locomotor activity, measured independently in each of the four quadrants of the open-field arena.**

| Left bottom area | | | | | | | | | | | | | | | | | |
| --- | --- | --- | --- | --- | --- | --- | --- | --- | --- | --- | --- | --- | --- | --- | --- | --- | --- |
|  | Pre | PD | | DN-1M | | DN-3Ms | | DN-6Ms | | DN-9Ms | | DN-12Ms | | DN-15Ms | | DN-18Ms | |
| L-Mut-DA-NPC (090025) | 2460.36 | 1662.63 | | 1533.63 | | 4694.06 | | 2133.57 | | 2432.33 | | 2519.32 | | 1585.52 | | 2155.52 | |
| L-Mut-DA-NPC (072289) | 2248.18 | 4810.81 | | 3995.13 | | Death | | Death | | Death | | Death | | Death | | Death | |
| L-Mut-DA-NPC (072197) | 1022.49 | 1726.89 | | 2103.10 | | 1664.30 | | 1537.30 | | 1055.59 | | 2386.72 | | 957.49 | | 576.91 | |
| L-DA-GC-NPC (081459) | 800.33 | 3638.30 | | 1884.72 | | 3536.87 | | 3721.62 | | 3323.16 | | 3935.47 | | 2965.13 | | 3844.38 | |
| L-DA-GC-NPC (061307) | 206.84 | 5150.42 | | 3734.63 | | 3328.90 | | 2107.34 | | 2655.22 | | 3234.30 | | 2036.24 | | 3081.28 | |
| L-DA-GC-NPC (091713) | 3596.3 | 5081.68 | | 103.77 | | 362.86 | | 287.42 | | 1399.00 | | 1289.76 | | 1640.07 | | 2273.44 | |
| Control (07649) | 1938.00 | 1513.35 | | 1660.69 | | 2533.67 | | 2834.00 | | 2788.42 | | 2663.03 | | 2514.65 | | 1573.37 | |
| Control (086259) | 3890.46 | 2790.36 | | 3612.48 | | 1559.86 | | 1564.70 | | 651.02 | | 1197.43 | | 835.24 | | 3102.64 | |
| Control (071475) | 3548.62 | 675.08 | | 807.67 | | 5261.19 | | 3592.79 | | 3709.14 | | 4103.37 | | 4636.27 | | ND | |
| PD (091489) | 3475.21 | 5400.00 | | 1823.86 | | ND | | 4423.02 | | 4494.39 | | 4249.05 | | 3417.78 | | 4492.23 | |
| PD (085175) | 2612.88 | 4278.41 | | 2664.13 | | 3178.28 | | 2498.80 | | 1051.05 | | 1427.96 | | 1293.59 | | ND | |
| PD (056507) | 2629.16 | 1975.71 | | 2283.15 | | 2527.19 | | 2198.26 | | 2326.83 | | 2262.06 | | 1783.52 | | ND | |
| Right bottom area | | | | | | | | | | | | | | | | | |
|  | Pre | | PD | | DN-1M | | DN-3Ms | | DN-6Ms | | DN-9Ms | | DN-12Ms | | DN-15Ms | | DN-18Ms |
| L-Mut-DA-NPC (090025) | 2919.79 | | 3737.37 | | 3866.4 | | 454.82 | | 3078.98 | | 2710.78 | | 2648.35 | | 2683.78 | | 2870.64 |
| L-Mut-DA-NPC (072289) | 2820.59 | | 589.19 | | 0 | | Death | | Death | | Death | | Death | | Death | | Death |
| L-Mut-DA-NPC (072197) | 4129.60 | | 3673.11 | | 3252.19 | | 3723.96 | | 3862.73 | | 4313.85 | | 3201.03 | | 4442.54 | | 4460.89 |
| L-DA-GC-NPC (081459) | 1378.91 | | 1704.64 | | 3123.39 | | 1059.59 | | 694.16 | | 1598.13 | | 1275.08 | | 1381.95 | | 1435.74 |
| L-DA-GC-NPC (061307) | 5193.16 | | 249.58 | | 1622.99 | | 2024.06 | | 1538.84 | | 1867.7 | | 1656.32 | | 2555.82 | | 2105.14 |
| L-DA-GC-NPC (091713) | 675.98 | | 222.99 | | 3476.98 | | 3431.93 | | 4081.01 | | 1660.63 | | 3498.93 | | 2029.56 | | 2051.08 |
| Control (07649) | 3337.37 | | 3875.11 | | 3687.22 | | 2669.14 | | 2269.24 | | 2342.24 | | 2463.5 | | 2491.29 | | 3644.28 |
| Control (086259) | 1083.78 | | 2349.72 | | 1751.02 | | 2861.86 | | 1832.40 | | 1463.70 | | 2334.10 | | 1096.13 | | 708.38 |
| Control (071475) | 1768.20 | | 2584.85 | | 3575.74 | | 109.64 | | 1746.28 | | 1533.43 | | 1094.13 | | 705.14 | | ND |
| PD (091489) | 1715.62 | | 0 | | 3576.18 | | ND | | 945.31 | | 905.64 | | 1653.95 | | 1982.25 | | 893.96 |
| PD (085175) | 1840.81 | | 957.36 | | 2301.27 | | 2023.09 | | 2338.97 | | 4641.88 | | 3838.47 | | 4052.69 | | ND |
| PD (056507) | 2265.00 | | 3391.19 | | 2836.74 | | 2761.90 | | 3154.92 | | 3090.02 | | 2931.97 | | 3455.66 | | ND |
| Left top area | | | | | | | | | | | | | | | | | |
|  | Pre | | PD | | DN-1M | | DN-3Ms | | DN-6Ms | | DN-9Ms | | DN-12Ms | | DN-15Ms | | DN-18Ms |
| L-Mut-DA-NPC (090025) | 19.85 | | 0 | | 0 | | 11.78 | | 76.91 | | 210.91 | | 46.18 | | 106.41 | | 72.91 |
| L-Mut-DA-NPC (072289) | 254.99 | | 0 | | 0 | | Death | | Death | | Death | | Death | | Death | | Death |
| L-Mut-DA-NPC (072197) | 0 | | 0 | | 0 | | 11.78 | | 0 | | 0 | | 0 | | 0 | | 0 |
| L-DA-GC-NPC (081459) | 2922.66 | | 0 | | 153.49 | | 11.78 | | 730.23 | | 409.78 | | 189.86 | | 330.73 | | 51.72 |
| L-DA-GC-NPC (061307) | 0 | | 0 | | 37.90 | | 0 | | 1695.30 | | 834.53 | | 672.31 | | 700.03 | | 211.31 |
| L-DA-GC-NPC (091713) | 1047.45 | | 53.09 | | 219.59 | | 11.78 | | 621.92 | | 1676.61 | | 420.29 | | 655.46 | | 114.95 |
| Control (07649) | 30.50 | | 0 | | 0 | | 121.55 | | 102.50 | | 143.81 | | 134.60 | | 57.79 | | 23.99 |
| Control (086259) | 331.97 | | 66.77 | | 7.97 | | 293.93 | | 1561.96 | | 2798.13 | | 2752.79 | | 1761.19 | | 782.55 |
| Control (071475) | 55.99 | | 1279.75 | | 559.73 | | 25.19 | | 0 | | 110.54 | | 95.86 | | 32.97 | | ND |
| PD (091489) | 115.28 | | 0 | | 0 | | ND | | 31.70 | | 0 | | 0.37 | | 0 | | 0 |
| PD (085175) | 406.94 | | 19.89 | | 324.56 | | 0 | | 233.73 | | 29.46 | | 27.03 | | 44.58 | | ND |
| PD (056507) | 189.56 | | 12.31 | | 107.64 | | 64.7 | | 46.61 | | 92.26 | | 43.38 | | 0 | | ND |
| Right top area | | | | | | | | | | | | | | | | | |
|  | Pre | | PD | | DN-1M | | DN-3Ms | | DN-6Ms | | DN-9Ms | | DN-12Ms | | DN-15Ms | | DN-18Ms |
| L-Mut-DA-NPC (090025) | 0 | | 0 | | 0 | | 0 | | 110.58 | | 24.02 | | 54.65 | | 148.98 | | 57.66 |
| L-Mut-DA-NPC (072289) | 76.24 | | 0 | | 0 | | Death | | Death | | Death | | Death | | Death | | Death |
| L-Mut-DA-NPC (072197) | 247.51 | | 0 | | 0 | | 0 | | 0 | | 0 | | 29.86 | | 0 | | 0 |
| L-DA-GC-NPC (081459) | 257.99 | | 2.14 | | 238.44 | | 213.01 | | 254.02 | | 68.97 | | 95.53 | | 132.57 | | 68.20 |
| L-DA-GC-NPC (061307) | 0 | | 0 | | 0 | | 0 | | 0 | | 42.58 | | 68.34 | | 0 | | 0 |
| L-DA-GC-NPC (091713) | 54.95 | | 42.24 | | 617.72 | | 226.46 | | 409.68 | | 663.80 | | 46.78 | | 279.25 | | 67.30 |
| Control (07649) | 46.11 | | 11.58 | | 52.12 | | 75.68 | | 80.58 | | 124.92 | | 122.86 | | 7.14 | | 0 |
| Control (086259) | 93.53 | | 114.61 | | 28.56 | | 684.38 | | 440.97 | | 447.45 | | 658.79 | | 502.20 | | 215.18 |
| Control (071475) | 27.23 | | 860.36 | | 411.78 | | 2.27 | | 0 | | 51.35 | | 61.59 | | 0 | | ND |
| PD (091489) | 93.89 | | 0 | | 0 | | ND | | 0 | | 0 | | 0.20 | | 0 | | 0 |
| PD (085175) | 539.41 | | 144.38 | | 110.08 | | 135.17 | | 331.63 | | 71.60 | | 102.17 | | 0 | | ND |
| PD (056507) | 316.32 | | 20.82 | | 172.51 | | 0 | | 0 | | 201.13 | | 78.71 | | 140.37 | | ND |

These values constitute the primary dataset underlying Figure 5C–F. Behavioral assessments were conducted at pre-MPTP baseline (Pre), post-MPTP lesion prior to transplantation (PD), and at quarterly intervals from 1 to 18 months post-grafting (DN-1M to DN-18M). Continuous, synchronous tracking across all four quadrants was performed throughout the entire experimental period to ensure spatially resolved activity quantification per quadrant. Monkey L-Mut-DA-NPC (072289) died at DN-2M, so no further behavioral data were collected for this animal beyond that time point. A value of 0 indicates absence of detectable spontaneous locomotor activity in the specified quadrant at the given time point. ND = Not Done. All the ND entries correspond to missing behavioral measurements were due to the scheduling conflicts during experimental execution.

**Table S7. Blood biochemistry analysis of monkeys after transplantation.**

| Animal ID | Group | ALT (U/L) | AST (U/L) | BUN (mM) | CRE (μM) | LDH (U/L) | AFP (ng/ml) | CEA (ng/ml) | NSE (ng/ml) | Timing |
| --- | --- | --- | --- | --- | --- | --- | --- | --- | --- | --- |
| 090025 | DA NPC1 | 5.49 | 2.37 | 6.71 | 131.23 | 42.42 | 1.98 | 2.84 | 0.22 | Pre-MPTP |
|  |  | 1.62 | 1.28 | 4.69 | 120.36 | 35.55 | 2.45 | 2.98 | 0.21 | Pre-grafting |
|  |  | 15.20 | 6.98 | 6.87 | 147.09 | 42.01 | 1.94 | 2.80 | 0.24 | Post-MPTP |
| 072197 | DA NPC1 | 8.63 | 4.19 | 4.35 | 140.98 | 50.60 | 1.85 | 2.74 | 0.18 | Post-grafting |
|  |  | 8.29 | 4.19 | 4.85 | 132.10 | 54.10 | 2.00 | 3.18 | 0.21 | Pre-grafting |
|  |  | 4.57 | 1.01 | 4.44 | 157.60 | 37.38 | 1.46 | 2.66 | 0.23 | Pre-MPTP |
| 081459 | DA NPC2 | 5.60 | 3.20 | 5.86 | 122.87 | 44.33 | 2.09 | 2.83 | 0.16 | Pre-MPTP |
|  |  | 1.99 | 1.62 | 5.66 | 108.27 | 40.39 | 1.73 | 2.76 | 0.19 | Pre-grafting |
|  |  | 3.09 | 15.42 | 6.59 | 159.31 | 44.33 | 1.84 | 2.82 | 0.27 | Post-MPTP |
| 061307 | DA NPC2 | 1.76 | 2.84 | 7.88 | 117.62 | 59.39 | 1.75 | 2.79 | 0.18 | Post-grafting |
|  |  | 1.45 | 1.10 | 5.19 | 115.19 | 42.89 | 1.90 | 3.09 | 0.32 | Pre-grafting |
|  |  | 3.67 | 1.89 | 6.93 | 184.74 | 54.83 | 1.91 | 3.00 | 0.24 | Pre-MPTP |
| 091713 | DA NPC2 | 5.60 | 2.42 | 3.23 | 109.85 | 46.54 | 2.03 | 3.22 | 0.25 | Pre-grafting |
|  |  | 7.57 | 3.11 | 5.23 | 106.47 | 50.09 | 2.20 | 3.07 | 0.33 | Pre-MPTP |
|  |  | 1.53 | 1.48 | 3.68 | 113.48 | 36.05 | 1.72 | 2.73 | 0.27 | Post-MPTP |
| 091489 | PD | 7.86 | 1.76 | 4.40 | 115.90 | 42.62 | 1.96 | 2.67 | 0.18 | Post-grafting |
|  |  | 22.88 | 21.86 | 5.74 | 130.51 | 48.69 | 1.83 | 2.51 | 0.20 | Pre-grafting |
|  |  | 31.78 | 8.64 | 6.09 | 98.08 | 62.40 | 2.00 | 2.98 | 0.21 | Pre-MPTP |
| 085175 | PD | 11.03 | 7.85 | 6.98 | 108.94 | 44.34 | 1.55 | 2.92 | 0.18 | Pre-grafting |
|  |  | 12.20 | 31.74 | 4.28 | 174.12 | 50.95 | 1.91 | 3.11 | 0.26 | Pre-MPTP |
|  |  | 8.98 | 19.70 | 5.60 | 130.74 | 37.79 | 1.72 | 2.62 | 0.22 | Post-MPTP |
| 056507 | PD | 14.58 | 1.98 | 4.72 | 100.72 | 40.09 | 2.14 | 2.97 | 0.21 | Post-grafting |
|  |  | 5.69 | 4.36 | 3.81 | 110.88 | 51.36 | 2.17 | 2.94 | 0.23 | Pre-grafting |
|  |  | 18.48 | 35.03 | 5.80 | 137.36 | 42.28 | 1.73 | 2.81 | 0.20 | Pre-grafting |
| 07649 | Control | 24.71 | 6.75 | 7.09 | 134.09 | 43.71 | 1.54 | 2.67 | 0.20 | Post-grafting |
| 086529 | Control | 12.60 | 10.43 | 5.35 | 116.77 | 40.77 | 1.61 | 2.55 | 0.26 | Post-grafting |
| Range | | 5 - 40 | 5 - 40 | 2.88 - 7.2 | 44 - 133 | 40 - 120 | < 10 | < 5 | < 15 | – |

Serum levels of liver function markers (ALT, AST), renal function markers (BUN, CRE), LDH, and tumor-associated antigens (AFP, CEA, NSE) were detected to assess systemic safety. All values retained within normal physiological ranges across all groups, indicating no evidence of toxicity or tumorigenicity. Data are shown as individual measurements at different time points (Pre-MPTP, Pre-grafting, Post-MPTP, Post-grafting). ALT: Alanine aminotransferase; AST: Aspartate aminotransferase; BUN: Blood urea nitrogen; CRE: Creatinine; LDH: Lactate dehydrogenase; AFP: Alpha-fetoprotein; CEA: Carcinoembryonic antigen; NSE: Neuron-specific enolase.

**Table S8. The H&Y scores blindly by two well-trained technicians.**

| Categories | 0 | 1 | 2 | 3 |
| --- | --- | --- | --- | --- |
| Facial expression | Absent | Mild/not persistently present | Moderate | Severe |
| Rest tremor | Absent | Mild/not persistently present | Moderate | Severe |
| Motor tremor | Absent | Mild/not persistently present | Moderate | Severe |
| Defense | Absent | Mild/not persistently present | Moderate | Severe |
| Gait | Absent | Mild/not persistently present | Moderate | Severe |
| Bradykinesia | Absent | Mild/not persistently present | Moderate | Severe |
| Balance/coordination | Absent | Mild/not persistently present | Moderate | Severe |
| Posture | Normal | Impaired | Frequent falling | No movement |
| Gross motor skills of upper limb | Normal | Slightly reduced | Reduced | No walking |
| Gross motor skills of lower limb | Normal | Slightly reduced | Reduced | No walking |

H&Y scores for evaluating Parkinson’s disease-related motor symptoms, as assessed independently by two trained technicians. Scores range from 0 (absent/no impairment) to 3 (severe/no function), with specific descriptors for each category and score level..
